# Psychological factors enhanced heterogeneous learning interactive graph knowledge tracing for understanding the learning process

**DOI:** 10.3389/fpsyg.2024.1359199

**Published:** 2024-05-10

**Authors:** Zhifeng Wang, Wanxuan Wu, Chunyan Zeng, Heng Luo, Jianwen Sun

**Affiliations:** ^1^Faculty of Artificial Intelligence in Education, Central China Normal University, Wuhan, China; ^2^CCNU Wollongong Joint Institute, Central China Normal University, Wuhan, China; ^3^Hubei Key Laboratory for High-Efficiency Utilization of Solar Energy and Operation Control of Energy Storage System, Hubei University of Technology, Wuhan, China

**Keywords:** psychological factors, knowledge tracing, Graph Neural Network, Item Response Theory, learning process

## Abstract

**Introduction:**

With the rapid expansion of online education, there is a burgeoning interest within the EdTech space to offer tailored learning experiences that cater to individual student's abilities and needs. Within this framework, knowledge tracing tasks have garnered considerable attention. The primary objective of knowledge tracing is to develop a model that assesses a student's proficiency in a particular skill based on their historical performance in exercises, enabling predictions regarding the likelihood of correct responses in future exercises. While existing knowledge tracing models often incorporate information such as students' exercise answering history and skill mastery level, they frequently overlook the students' mental states during the learning process.

**Methods:**

This paper addresses this gap by introducing a novel psychological factors-enhanced heterogeneous learning interactive graph knowledge tracing model (Psy-KT). This model delineates the interactions among students, exercises, and skills through a heterogeneous graph, supplementing it with four psychological factors that capture students' mental states during the learning process: frustration level, confusion level, concentration level, and boredom level. In the modeling of students' learning processes, we incorporate the forgetting curve and construct relevant cognitive parameters from the features. Additionally, we employ the Item Response Theory (IRT) model to predict students' performance in answering exercises at the subsequent time step. This model not only delves into the psychological aspects of students during the learning process but also integrates the simulation of forgetting, a natural phenomenon in the learning journey. The inclusion of cognitive parameters enhances the description of changes in students' abilities throughout the learning process. This dual focus allows for a more comprehensive understanding of students' learning behaviors while providing a high level of interpretability for the model.

**Results and discussion:**

Empirical validation of the Psy-KT model is conducted using four publicly available datasets, demonstrating its superior performance in predicting students' future performance. Through rigorous experimentation, the integration of psychological and forgetting factors in the Psy-KT model not only improves predictive accuracy but also enables educators to offer more targeted tutoring and advice, enhancing the overall efficacy of the learning experience.

## 1 Introduction

In the contemporary landscape of education, the prevalence of online education platforms has witnessed a substantial increase (Adedoyin and Soykan, 2023). These platforms, initially designed for college students acquiring various skills, have evolved into comprehensive systems catering to primary and high school students, augmenting their understanding of textbook knowledge (Wang et al., 2023c). A growing number of students are opting for diverse education platforms to acquire knowledge and refine their skills in this era of information-driven education.

Within the dynamic realm of online education, the imperative lies in optimizing learning modes to align with the evolving needs of students. For instance, subject-specific education modes can be enhanced by tailoring them to accommodate the Ebbinghaus forgetting curve, thus facilitating the development of distinct learning modes tailored to different subjects (Su et al., 2023). Moreover, recognizing the inherent diversity in students' comprehension levels for various types of knowledge, coupled with disparate learning methods and varying degrees of practice, underscores the need for online education platforms to prioritize personalized student development (Wang et al., 2023b).

A pivotal aspect in this context is the knowledge tracing task, a mechanism designed to predict a student's proficiency in handling subsequent tasks by modeling the evolving state of their knowledge during the skill-learning process. Originating in 1994, this task was initially conceptualized using Hidden Markov Models (Corbett and Anderson, 1995). Subsequent research ushered in the era of deep learning models for knowledge tracing, exemplified by the Deep Knowledge Tracing model (DKT; Piech et al., 2015) and the Convolutional Knowledge Tracing model (CKT; Shen et al., 2020), both markedly outperforming traditional knowledge tracing models.

In 2019, Nakagawa et al. introduced a paradigm shift by incorporating graph structures into the knowledge tracing task, resulting in the Graph Neural Network based Knowledge Tracing model (GKT; Nakagawa et al., 2019). This innovative approach not only validated the efficacy of graph structures but also offered a fresh perspective on constructing the relationships between skills and exercises. Subsequent research endeavors have witnessed further enhancements in the performance of knowledge tracing models based on graph structures (Li and Wang, 2023b), thereby catalyzing the advancement and innovation in this field. This progression not only attests to the continuous evolution of knowledge tracing techniques but also underscores their instrumental role in shaping the future of educational technology.

### 1.1 The motivation

The psychological state of students plays a pivotal role in shaping their learning processes and, consequently, influencing their educational outcomes. Past research has consistently underscored the impact of students' perceptions and experiences on their learning performance (Burden, 1994). Crucial components of students' psychology include their emotional states, motivation levels, and interest in the subject. The ability of students to actively engage in their learning and effectively navigate challenges encountered during their studies directly correlates with their overall learning outcomes.

Research conducted by Obergriesser and Stoeger has highlighted the significance of self-efficacy (an individual's confidence in successfully completing a task) and anxiety in determining students' likelihood of underachievement (Obergriesser and Stoeger, 2015). Notably, interventions tailored to address these psychological factors have been shown to positively impact students across varying academic abilities (Dignath and Büttner, 2008). Research by Matthew Owens and his team has shown that anxiety and working memory capacity (WMC) interact, influencing cognitive test outcomes differently depending on the individual's WMC. Individuals with low WMC experience a decline in test scores as anxiety increases, whereas those with high WMC see the opposite effect (Owens et al., 2014). These findings highlight the significant role of psychological factors like self-efficacy and anxiety not just in influencing academic performance but also in affecting diverse student populations differently. Additionally, these factors do not function independently; rather, they interplay, collectively shaping students' performance and development. Recognizing the pivotal role of students' psychological states in the learning process, it becomes imperative to deepen our understanding and attentiveness to this aspect for the development of effective teaching strategies and the enhancement of education quality.

Furthermore, the interaction between learners and educational resources is pivotal in formative assessments. This interaction not only enables teachers to gauge students' progress and needs but also supports learners in self-assessment and in fine-tuning their study strategies. However, much of the prior research has emphasized the influence of students' cognition and experiences on their academic outcomes, primarily by enhancing knowledge tracing models through a representation of the relationships between skills and exercises (Lyu et al., 2022). This optimization has progressed from initially utilizing Long Short-Term Memory (LSTM) networks to analyze answer sequences to currently employing graph structures that map out the intricate dynamics between exercises and skills (Li and Wang, 2023a). Additionally, there is ongoing work to improve the representation of students' skill mastery, encapsulating it within hidden states of student knowledge (Wang et al., 2023a). Modeling the complex heterogeneous interactions of learner, exercise, and knowledge throughout the learning process can significantly improve our understanding and optimization of educational practices, thereby boosting learning efficiency.

This paper considers students' psychological states during exercise answering as noteworthy features to be considered. Therefore, building upon previous research, it incorporates these psychological factors into knowledge tracing tasks to pursue a more comprehensive description of students' states. In real-world educational settings, the pursuit of efficient educational practices often aligns with the desire for credible and convincing outcomes. The inclusion of psychological factors in the model not only enhances its interpretability but also renders educational interventions more effective, catering to the diverse needs of students. Consequently, this paper advocates for the integration of students' psychological factors into knowledge tracing models to enrich their descriptive capacity and elevate their practical efficacy in educational contexts.

### 1.2 Our solution

To enhance the optimization of our model, this paper introduces the integration of psychological factors into the knowledge tracing framework to provide a nuanced depiction of students' mental states during online learning.

Firstly, our approach begins by examining the complex heterogeneous relationship among “student-exercise-skill.” To capture the intricate interconnections among “student-exercise-skill,” we construct a heterogeneous graph, employing a graph convolutional network specifically designed for such structures. This network efficiently integrates data from both nodes and their neighbors, enabling a comprehensive understanding of the local interactions among students, exercises, and skills. The convolutional operations on this heterogeneous graph allow the model to discern interactions at multiple levels, substantially improving its capacity to represent data.

Secondly, recognizing the natural occurrence of forgetting during online learning, we incorporate a forgetting function into the model to simulate the forgetting behavior during learning process. This inclusion enables the model to adapt more effectively to the dynamic nature of students' long-term learning on the one hand and provide a quantitative explanation for forgetting behaviors in online learning on the other hand.

Thirdly, we delineate students' knowledge states by constructing a directed sequential graph that abstracts their learning sequences. This graph captures the temporal relationships within students' processes of answering exercises, with each step depicted through nodes and edges. We introduce a graph gating neural network to handle the complexities of these temporal relationships. This network utilizes both the structural and temporal characteristics of the graph to dynamically adjust information transfer at each node, thus enhancing the model's ability to monitor and adapt to changes in a student's knowledge state during the learning sequence.Furthermore, during this analytical phase, the exercise sequences are not only considered in isolation but are also enriched with data on students' psychological factors, such as emotions and cognition. This comprehensive approach allows the model to more accurately reflect the students' learning states, taking into account psychological impacts on learning behaviors. This not only deepens the understanding of the learning process but also enriches the model's ability to predict and support students' learning needs effectively.

Finally, we employ the Item Response Theory as the predictive layer for learning performance, which generates accurate predictions of students' performance on subsequent exercises. IRT is particularly effective due to its commendable quantitative accuracy in forecasting learning outcomes and its compatibility with the forgetting function. Furthermore, the cognitive parameters within the IRT model provide significant insights into the cognitive attributes of both learners and learning resources, enhancing the model's explanatory power.

Through these methodologies, we aim to create a robust, comprehensive model that not only predicts learning outcomes with high accuracy but also incorporates a nuanced understanding of the psychological factors influencing students during online learning. This integrated approach ensures a deeper insight into the educational process, significantly contributing to the fields of educational technology and psychology.

### 1.3 Summary of contribution

This paper introduces several significant contributions to the field of knowledge tracing through the development of the Psy-KT model, outlined as follows:

1. **Comprehensive integration of psychological factors:** The Psy-KT model introduces a nuanced approach by incorporating psychological aspects such as frustration, concentration, confusion, and boredom levels into the learning process analysis. This incorporation aims to provide a more comprehensive understanding of students' emotional and cognitive states during learning. Alongside psychological factors, the model includes a forgetting curve to account for skill decay over time, thus addressing a critical aspect of long-term learning retention. It also incorporates exercise difficulty characteristics and leverages the Item Response Theory to enhance the accuracy of predicting students' future responses, thereby significantly improving the model's predictive performance.
2. **Innovative use of a heterogeneous learning interactive graph:** The Psy-KT model employs a heterogeneous learning interactive graph that captures the complex interrelations among students, exercises, and skills within a learning environment. This graph provides a dynamic and detailed representation of students' progression through exercise sequences, depicted through nodes and edges that map each response step. To manage the temporal complexities embedded in these interactions, a graph gating neural network is introduced. This network is specifically designed to utilize the graph's structural and temporal data, dynamically adjusting information transfer at each node. Its adaptability is crucial for accurately reflecting changes in the learning process and responding to individual student needs.
3. **Empirical validation and superior performance:** Through extensive experimentation using four publicly available datasets, the Psy-KT model has demonstrated superior performance compared to existing knowledge tracing models. The enhancement in performance is largely attributed to the model's unique features, including the integration of psychological factors, the application of a forgetting function, and the use of IRT for predictions. These features collectively improve the model's adaptability and depth of understanding regarding the complexities of student learning processes. This leads to a marked improvement in performance, highlighting the effectiveness of combining psychological insights with advanced data modeling techniques in educational settings.

## 2 Related work

This section aims to provide an overview of pertinent models in the related domains. The discussion will commence with an exploration of models related to Graph Neural Networks, followed by an examination of current popular models employed for cognitive diagnostics. The following section will culminate with a review of models that have demonstrated noteworthy results in the realm of knowledge tracing.

### 2.1 Graph Neural Networks

A graph structure, representing non-Euclidean data structures like transportation networks or chemical molecules, is characterized by nodes and edges denoted as *G* = (*V, E*), where *V* is the set of nodes, and *E* is the set of edges (Wu et al., 2021). Beyond nodes and edges, graphs can incorporate attributes such as weights, orientations, and labels, enriching their applicability to real-world problems. For instance, in a social network, a graph can depict users and their connections, with nodes representing users, edges denoting relationships, and weights indicating connection frequencies.

To extend deep learning to graph-structured data, Gori et al. introduced the Graph Neural Network (GNN), designed to process graph structures directly (Gori et al., 2005). Their experiments showcased the applicability of GNN to various practical graph structures, including directed, undirected, and cyclic graphs.

Building upon the GNN foundation, subsequent studies drew inspiration from Convolutional Neural Networks (CNNs), leading to the emergence of graph convolutional networks. These models can generally be categorized into spectral-based and spatial-based approaches (Wu et al., 2021). Spectral-based methods leverage the eigenvalue decomposition of the Laplace matrix to introduce convolution operations in the frequency domain. Exemplary models include those proposed by Bruna et al. (2014) and Defferrard et al. (2016), proficient in capturing global information across entire graph structures. In contrast, spatial-based methods focus on the local structure between a node and its neighbors. Notable models in this category include those introduced by Atwood and Towsley (2016) and Niepert et al. (2016), which aggregate information from neighboring nodes to model the local context of each node.

The evolution of Graph Neural Networks has marked a prominent research direction in deep learning. Models like Graph Sample and Aggregation (GraphSAGE; Hamilton et al., 2017), and Graph Attention Network (GAT; Veličković et al., 2018), have expanded the frontiers of graph data processing. Innovations such as attention mechanisms and sampling strategies in these models enhance the capability for representation learning in graph data, providing a robust tool for diverse applications in the knowledge tracing field.

### 2.2 Cognitive diagnosis

Cognitive diagnosis aims to delve into students' cognitive states during the learning process to evaluate their learning abilities and offer personalized support. Representative models, rooted in previous research, encompass the Item Response Theory and the Deterministic Input, Noisy “And” model (DINA). The former synthesizes students' abilities and the exercise parameters in the learning process, while the latter delineates the specific knowledge mastery state of students. The interplay of these two models has propelled the diversification and evolution of cognitive diagnostic theories.

1. **Item Response Theory:** Item Response Theory, also known as Latent Trait Theory, posits that individuals possess latent traits, suggesting a close connection between subjects' response scores on test items and these latent traits (Embretson and Reise, 2013). In the context of knowledge tracing tasks, IRT is grounded in the relationship between student ability and the probability of correct answers.

Assuming a student's ability is denoted by θ, the probability function *P*(θ) evolves over time, representing the likelihood that a student with a certain ability level will answer an exercise correctly. This probability is calculated according to Equation (1) (Moustaki and Knott, 2000).


(1) $$\begin{array}{l} P\left(\theta \right)=\frac{c*\left(1-c\right)}{1+e^{-\alpha \left(\theta -\beta \right)}} \end{array}$$

In this equation, α represents the differentiation of the exercise, indicating the exercise's ability to discern between students' levels. β denotes the difficulty of the exercise, where an increase in β necessitates higher θ for a higher probability of a correct answer. The parameter *c* is the guessing parameter, signifying the probability that a student can answer an exercise correctly by guessing. For the purposes of this paper, *c* is set to 0, indicating that students cannot answer the exercise correctly by guessing alone. This formulation provides a nuanced understanding of how student ability, exercise differentiation, difficulty, and guessing interact in the context of IRT for knowledge tracing tasks.

2. **Deterministic Input, Noisy “And” model:** The DINA model serves as a discrete cognitive diagnostic model, conceptualizing a student as a multidimensional vector of knowledge mastery, diagnosed based on their actual response outcomes (de la Torre, 2009). The model introduces the potential response (η_*ij*_) of student *i* in exercise *j*, as defined by Equation (2).

(2) $$\begin{array}{l} \eta_{ij}=\overset{K}{\underset{k=1}{\prod }}\alpha_{ik}^{q_{j}k} \end{array}$$

Here, α_*ik*_ denotes the student *i*'s mastery of skill *k*. If η_*ij*_ = 1, it indicates a correct answer, implying mastery of all skills in exercise *j*. Conversely, if η_*ij*_ = 0, it signals an incorrect answer, signifying a lack of mastery in at least one skill within exercise *j*. The DINA model integrates the question-skill correlation matrix (*Q*) and the student answer matrix (*X*) to model a student's response. It introduces test item parameters, namely (*slip, guess*), resulting in Equation (3) to estimate the probability [*P*_*j*_(α_*i*_)] of a student's ability to answer exercise *j* correctly (de la Torre, 2009).


(3) $$\begin{array}{l} P_{j}\left(\alpha_{i}\right)=P\left(X_{ij}=1|\alpha_{i}\right)=g_{j}^{1-\eta_{ij}}{\left(1-s_{j}\right)}^{\eta_{ij}} \end{array}$$

In addition to these cognitive diagnosis models, other notable models like the Grade Response Model (GRM; Samejima, 1969), and the Fuzzy Cognitive Diagnosis Model (FuzzyCDM; Liu et al., 2018), have been developed for static characterization of students' abilities, offering deeper insights into their knowledge states. However, in the dynamic process of teaching and learning, where students are consistently engaging with exercises, their cognitive levels are in a state of flux. To address this dynamic nature, it is imperative to continuously update students' states and employ a dynamic approach for a more robust and accurate assessment process.

### 2.3 Knowledge tracing

In the evolution of knowledge tracing tasks, models have primarily fallen into two categories: traditional methods and deep learning methods. This section exclusively focuses on the latter, delving into the advancements brought about by deep learning in the context of knowledge tracing.

1. **DKT:** The integration of deep learning into knowledge tracing tasks was pioneered by Piech et al. with the introduction of Deep Knowledge Tracing, a Recurrent Neural Network (RNN) based model (Piech et al., 2015). DKT deploys a substantial number of neurons to capture temporal dynamic structures, utilizing a sequence of historical student interactions as input. This approach enables the model to dynamically learn from evolving student knowledge states during the learning process. The model transforms sequences of students' historical interactions into corresponding output sequences by navigating through a series of hidden knowledge states, ultimately providing probabilities for correct exercise responses. While DKT marked a significant advancement over traditional knowledge tracing models, it presents certain limitations, such as neglecting the phenomenon of forgetting during the learning process and exhibiting poor interpretability. Subsequent research endeavors have sought to address these shortcomings and enhance the overall performance of DKT. Several research teams have engaged in iterative improvements and optimizations in their subsequent works to refine and build upon the foundation laid by DKT.
2. **DKT+:** Due to the limitations of the algorithm used in DKT, it fails to consider long-term historical data, resulting in fluctuation phenomena where a student's latent state does not gradually increase or decrease over time but experiences sudden spikes or drops. Another issue in DKT is the inability to reconstruct input information, where a student performs poorly on exercises involving skill *s*_*i*_, yet the model predicts a high level of mastery for skill *s*_*i*_. The DKT+ model is proposed to address these issues by incorporating three regularization terms into the loss function of DKT (Yeung and Yeung, 2018). These three regularization terms are reconstruction error *r* and fluctuation measures ω_1_ and ω_2_.
3. **KPT:** The Knowledge Proficiency Tracing (KPT) model is constructed based on Probabilistic Matrix Factorization (PMF; Huang et al., 2020). This model associates exercises with skill vectors, establishing a correspondence between the two. The KPT model uses PMF technology to model students' answering behaviors, inferring their proficiency level for each skill.
4. **AKT:** The Attention-based Knowledge Tracing (AKT) model introduces attention mechanisms and assumes that a student's learning process is transient, with knowledge decaying over time (Ghosh et al., 2020). This model comprises four modules: embedding based on the Rasch model, exercise encoder, skill encoder, and knowledge retriever. AKT not only captures global relationships without considering the length of answer sequences but also enhances interpretability by integrating the Rasch psychometric model.
5. **DKVMN:** Zhang et al. introduced the Dynamic Key-Value Memory Network (DKVMN) model (Zhang et al., 2017). This model employs a static matrix to store knowledge skills and a dynamic matrix to store and update students' states. By leveraging the relationships between skills, the DKVMN model provides a direct output indicating a student's mastery level for each skill. Despite its strengths, it is noteworthy that the DKVMN network falls short in capturing long-term dependencies within sequences.
6. **GKT:** Nakagawa et al. pioneered the incorporation of graph structures into the knowledge tracing model, presenting the Graph-based Knowledge Tracing model (Nakagawa et al., 2019). In GKT, the relationships between knowledge points are depicted by a directed graph denoted as *G* = *V, E, A*, where *V* signifies the set of nodes, *E* signifies the set of directed edges, and *A* signifies the weight of each dependency. When updating the network model based on the graph structure using a multilayer perceptron, consideration is given not only to the state of the node itself but also to the state of a specified number of neighboring nodes. Subsequently, the updated embedded representation is employed to predict the student's performance at the next time step.
7. **GIKT:** Yang et al. introduced the Graph-Based Interaction Model (GIKT) as a novel approach to knowledge tracing (Yang et al., 2021). This model employs a Graph Convolutional Neural Network to facilitate the convergence of exercise and skill representations within an exercise-skill relationship graph. Additionally, a recursive layer is incorporated to enhance the model's capability to capture time-series variations and long-term dependencies in knowledge states. The GIKT model integrates two essential modules for improved predictive accuracy. Firstly, the Historical Recap module is designed to select the most pertinent hidden exercises from the historical data concerning the current exercise. Secondly, the Interaction module enables a two-way interaction among the student's current state, relevant historical exercises, the target exercise, and the skill prediction. This interactive mechanism significantly contributes to the final prediction, enhancing the model's overall predictive performance.
8. **SGKT:** Wu et al. introduced the Session Graph-Based Knowledge Tracing (SGKT) model as outlined in their work (Wu et al., 2022). The SGKT model employs a meticulously crafted heterogeneous graph that encompasses the elements “student-skill-exercise.” Through the application of convolutional neural networks, embedded representations of skills and exercises are derived from this graph. An innovative feature of SGKT lies in its inclusion of a forgetting mechanism, strategically integrated to simulate the phenomenon of forgetting within the learning process. In addition to the convolutional neural networks, SGKT utilizes a gated Graph Neural Network. This component plays a crucial role in extracting the student's hidden knowledge state, contributing to the comprehensive understanding of the student's learning trajectory. The final prediction is accomplished by combining the embedding representations of skills and exercises, reflecting the model's holistic approach to knowledge tracing.

The evolution of knowledge tracing tasks has witnessed remarkable advancements fueled by the integration of deep learning methodologies. Researchers have actively explored the fusion of neural networks and graph structures, striving for enhanced precision in tracking students' evolving knowledge states. Noteworthy achievements have been made by these models, particularly in accounting for the temporal dynamics inherent in the learning process, the intricate exercise-skill relationships graph, and the sequences of historical student interactions. Despite these strides, challenges persist within the knowledge tracing landscape. Issues such as model interpretability, treatment of long-term dependencies, and the accurate representation of the forgetting phenomenon during learning are focal points demanding further attention and refinement. Addressing these challenges is crucial for the continued advancement of knowledge tracing models in the realm of education.

## 3 Problem definition

### 3.1 Notations and definitions

This section rigorously provides the formal definitions of the psychological factors and interactive heterogeneous graphs involved in knowledge tracing tasks, and establishes a comprehensive set of mathematical notations used throughout the paper, as summarized in Table 1.

**Table 1:** Definitions of mathematical notation used in this paper.

**Notations**	**Descriptions**
*S, E, K*	A set of students, a set of exercises, and a set of skills
*X, P*	Answer records for a particular student, partial answer records
*s*_*i*_, *e*_*j*_, *k*_*q*_	Students number *i*, exercises number *j*, skills number *q*
*n*_*t*_	An exercise record answered by a student at a time step
*at*_*t*_	Time spent on exercise answered by a student at a time step
*fru*_*t*_	The effect of average anxiety level on students at a time step
*conf*_*t*_	The effect of average confusion on students at a time step
*conc*_*t*_	The effect of average concentration on students at a time step
*bor*_*t*_	The effect of average boredom on students at a time step
*a*_*t*_	Students' answers to exercises at a time step
*T*	Latest timestamp
*p*_*t*_	Probability of a student can correctly answer the next given exercise at timestamp *t*
*V, E*	The set of nodes and the set of edges in the SEK-HLIG
*r*_*se*_	Relationship between students and exercises
*r*_*ek*_	Relationship between exercises and skills
*m*(*x*)	Degree of memorization of a given knowledge skill
*z*_*i*_	Feature information of the *i*th node in the SEK-HLIG
$ẽ,\tilde{k}$	GCN outputs high-dimensional feature information of exercises and skills
*r*_*i*_	Node information in the SE-SG
*g*_*i*_	Student's state representation as input to GRU
*h*_*i*_	Student's hidden knowledge state
${\tilde{h}}_{i}$	Student's hidden knowledge state with added psychological features
Ê_*i*_	New student answer state processed by forgetting curve
*d*_*i*_	Exercise difficulty

#### 3.1.1 Definition of psychological factors

Recognizing the pivotal role of students' psychological states in the learning process, this paper deems it essential to integrate relevant features into the knowledge tracing model. The specific definitions are expounded below.

**Definition 1 (The frustration level):** The frustration level is an emotional indicator denoted as *fru*, reflecting the emotional state of the student during an exercise. This encompasses feelings of nervousness, worry, and uneasiness. The frustration level is a critical factor influencing a student's performance and motivation. Students experiencing high frustration levels may encounter learning disabilities, whereas moderate frustration levels could positively impact concentration and performance.

**Definition 2 (The confusion level):** The confusion level serves as a cognitive indicator denoted by *conf*, representing a student's ability to comprehend or solve an exercise. Throughout the learning process, students may grapple with confusion, and the extent of this confusion significantly influences their progress and learning experience.

**Definition 3 (The concentration level):** The concentration level is an attention indicator denoted as *conc*, gauging a student's ability to focus on an exercise. The concentration level holds substantial importance in the learning context. Higher levels of concentration facilitate enhanced knowledge absorption, while distractions may lead to diminished learning outcomes.

**Definition 4 (The boredom level):** The boredom level, denoted as *bor*, serves as an emotional indicator, revealing the degree to which students experience boredom or lack intrinsic motivation when engaging in exercises. Boredom in the learning context can lead to diminished motivation and suboptimal learning outcomes.

Research findings indicate that students' frustration levels and concentration positively correlate with learning outcomes. Conversely, boredom exhibits a weak negative correlation with learning outcomes, akin to the relationship observed between the level of confusion and boredom (Pardos et al., 2013). These identified psychological factors not only impact students' performance but also significantly influence their overall learning experience and motivation.

Integrating these psychological factors into the knowledge tracing model enhances our understanding of their effects on students' learning outcomes. This incorporation contributes to the overarching objective of personalized education, rendering the knowledge tracing model more comprehensive in its analysis and predictive capabilities.

**Definition 5 (Forgetting factor):** In the context of knowledge tracing tasks, forgetting is the gradual loss of previously acquired knowledge or skills over time, constituting a fundamental psychological phenomenon. This paper introduces the forgetting factor to simulate the dynamic nature of human learning and memory, thereby enhancing the model's ability to replicate the phenomenon of memory decay and improving its interpretability. The forgetting function (Chen et al., 2021), is expressed as in Equation (4).


(4) $$\begin{array}{l} m\left(x\right)=a\cdot {exp}^{-b\cdot x}+c \end{array}$$

Here, the parameters *a*, *b*, and *c* are fitting parameters, while *x* represents the time interval in days between the initial time and the current time. The function *m*(*x*) signifies the present degree of memory retention for a specific knowledge point. Lower values of *m*(*x*) indicate a higher degree of forgetting, with students experiencing a rapid forgetting rate in the initial stages, followed by a gradual slowing of the forgetting process.

Moreover, this paper posits that the psychological factors of students during the learning process can influence the time taken to answer an exercise. For instance, decreased concentration may lead to increased time spent comprehending the exercise. Therefore, this paper integrates the time *at*_*t*_ spent by the student to answer the exercise with the psychological factors during the exercise answering process. This synthesis is employed to characterize the student's hidden knowledge state in the scope of this paper's work.

#### 3.1.2 Definition of Heterogeneous Learning Interactive Graph

**Definition 6 (Student-Exercise-Skill Heterogeneous Learning Interactive Graph (SEK-HLIG)):** In the processing of students' exercise-answer sequences, we construct a heterogeneous learning interactive graph denoted as *HLIG* = {*V*; *E*}, representing the relationships between “student-exercise-skill.” The set of nodes *V* includes three types: student *s*, exercise *e*, and skill *k*, denoted as *V* = {*s, e, k*}. The set of edges *E* consists of two types: *r*_*se*_, representing the relationship between a student and an exercise, and *r*_*ek*_, representing the relationship between an exercise and a skill. Formally, *E* = {*r*_*se*_, *r*_*ek*_}. The edge *r*_*se*_ corresponds to the exercise that a student answered, while *r*_*ek*_ corresponds to the skill associated with the exercise.

**Figure 1:**
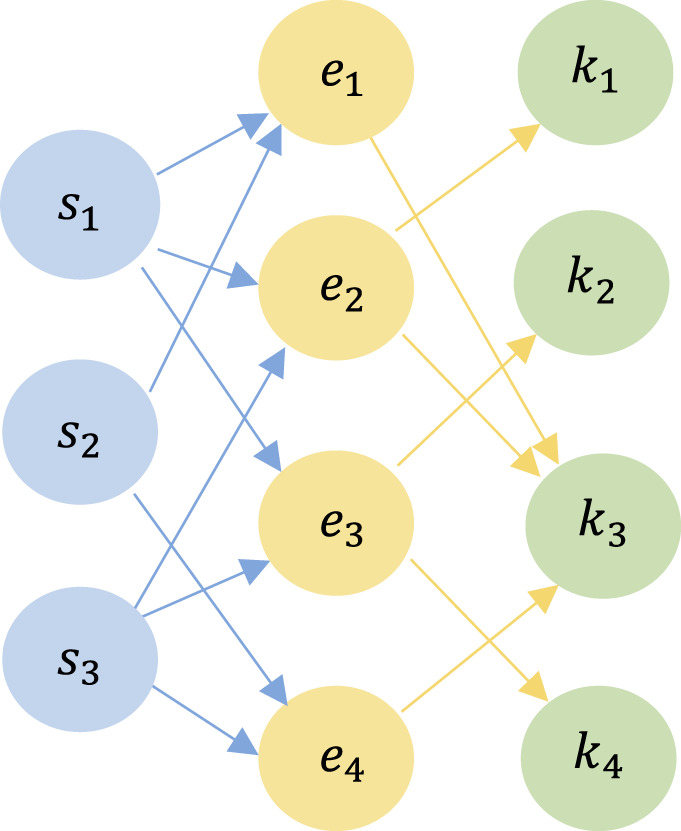
An example of a Heterogeneous Learning Interactive Graph with three student nodes, four exercise nodes, and four skill nodes. Two types of edges connect the three types of nodes, representing the corresponding relationships.

To illustrate, consider an example of an SEK-HLIG, depicted in Figure 1. In this graph, the set of students is {*s*_1_, *s*_2_, *s*_3_}, exercises are {*e*_1_, *e*_2_, *e*_3_, *e*_4_}, and skills are {*k*_1_, *k*_2_, *k*_3_, *k*_4_}. Collectively, these nodes form the set of all nodes *V* = {*s*_1_, *s*_2_, *s*_3_, *e*_1_, *e*_2_, *e*_3_, *e*_4_, *k*_1_, *k*_2_, *k*_3_, *k*_4_}. For instance, student *s*_1_ answered exercises *e*_1_, *e*_2_, and *e*_3_. Exercise *e*_1_ is associated with skill *k*_1_, while exercise *e*_2_ is associated with skills *k*_1_ and *k*_3_, and exercise *e*_3_ is associated with skills *k*_2_ and *k*_4_.

**Definition 7 (Student-Exercise Sequential Graph (SE-SG)):** In the context of a student's individual instances of answering exercises, we abstract the student's response sequence into a Student-Exercise Sequential Graph, denoted as *SG* = {*e*^*A*^, *P*}. Here, $e^{A}=\left\{e_{i}^{a}|1\leq i\leq n\right\}$ represents the answering situation of the *i*-th exercise, and *P*⊆*X* denotes the student's partial answer sequence.

**Figure 2:**
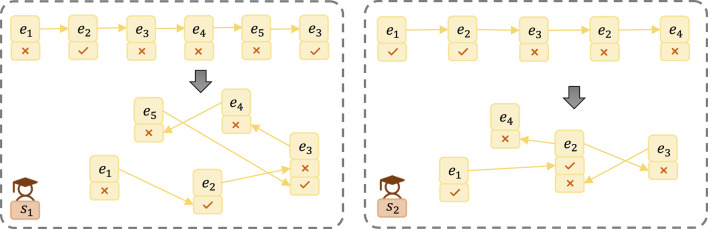
Example of a Student-Exercise Sequential Graph abstracting the sequence of student answers into a graph structure, facilitating direct observation of sequential relationships between exercises and their corresponding answers.

To illustrate this concept, consider the example of an SE-SG shown in Figure 2. The answer sequence is transformed into a graph structure, revealing the sequential relationships between exercises and corresponding answers. For instance, Student *s*_1_ exhibits an answer sequence of {*e*_1_, *e*_2_, *e*_3_, *e*_4_, *e*_5_, *e*_3_}, with corresponding answers being wrong, correct, wrong, wrong, wrong, and correct. Similarly, Student *s*_2_ demonstrates an answer sequence of {*e*_1_, *e*_2_, *e*_3_, *e*_2_, *e*_4_}, with corresponding answers being correct, correct, wrong, wrong, wrong.

### 3.2 Problem formulation

In the Psy-KT model, the goal is to predict the future learning performance *p*_*t*+1_ and track students' evolving mastery of skills over time 1 to *t*, based on student records *s*_*t*_, exercise records *e*_*t*_, answering records *a*_*t*_, psychological factors *psy*_*t*_ = {*fru*_*t*_, *con*_*t*_, *conf*_*t*_, *bor*_*t*_}, and time spent records *at*_*t*_. The objective is to predict the probability *p*_*t*+1_ of correctly answering a new exercise *e*_*t*+1_, enabling the development of personalized learning strategies to enhance student's learning efficiency. Unlike traditional knowledge tracing tasks that focus solely on the relationship between exercises and skills, this work integrates psychological factors experienced by students during exercise answering, thereby enhancing the knowledge tracing task. The model incorporates Item Response Theory to predict learning performance, improving interpretability.

The task is formally defined for a set of *N* students, denoted as *S* = {*s*_1_, *s*_2_, *s*_3_, …, *s*_*N*_}. The *n*−*th* student's learning record is represented as *X* = {*n*_1_, *n*_2_, *n*_3_, …, *n*_*T*_}, with *T* indicating the latest timestamp. A student's answer record at timestamp *t* is denoted as *n*_*t*_ = {*e*_*t*_, *k*_*t*_, *at*_*t*_, *psy*_*t*_, *a*_*t*_}, where:

- *e*_*t*_∈{*e*_1_, *e*_2_, *e*_3_, …, *e*_*m*_} represents the exercises the student answered at timestamp *t*.
- *k*_*t*_∈{*k*_1_, *k*_2_, *k*_3_, …, *k*_*p*_} denotes the skill associated with the exercise.
- *at*_*t*_ denotes the time spent answering the exercise.
- *psy*_*t*_ = {*fru*_*t*_, *con*_*t*_, *conf*_*t*_, *bor*_*t*_} is a set of psychological factors experienced by the student during the exercise, where *fru*_*t*_∈[0, 1] represents the average level of frustration, *conf*_*t*_∈[0, 1] represents the average level of confusion, *conc*_*t*_∈[0, 1] represents the average level of concentration, and *bor*_*t*_∈[0, 1] represents the average level of boredom.
- *a*_*t*_∈{0, 1} indicates whether the student answered the exercise correctly, with 0 indicating an incorrect answer and 1 indicating a correct answer.

## 4 The Psy-KT model

### 4.1 Model overview

**Figure 3:**
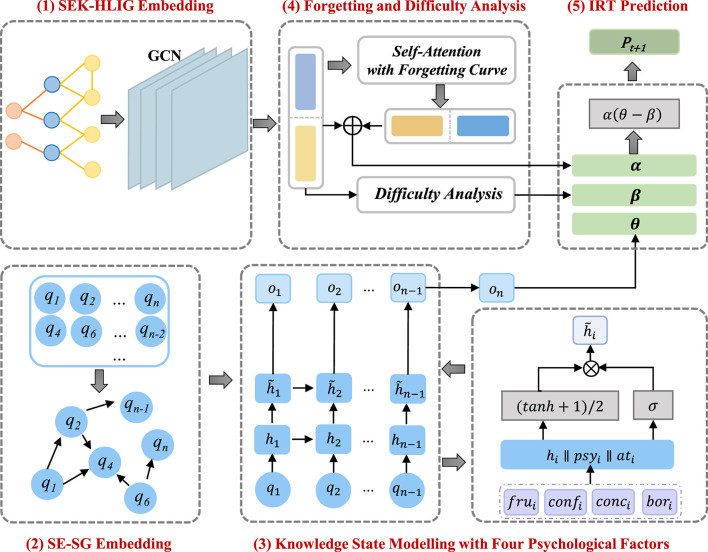
Schematic structure of the Psy-KT model network model. The model comprises five components, namely (1) SEK-HLIG Embedding Module, (2) SE-SG Embedding Module, (3) Knowledge State Modeling Module, (4) Forgetting and Difficulty Analysis Module, and (5) IRT Prediction Module.

This section provides a detailed overview of the Psy-KT model. Figure 3 illustrates the structural components of the proposed model, which encompasses five modules: (1) SEK-HLIG Embedding Module, (2) SE-SG Embedding Module, (3) Knowledge State Modeling Module, (4) Forgetting and Difficulty Analysis Module, and (5) IRT Prediction Module.

Upon inputting students' answer sequences into the model, the SEK-HLIG Embedding Module employs a Graph Convolutional Network (GCN) to learn the relationships between and within exercises and skills in the SEK-HLIG. Simultaneously, the Gated Graph Neural Network (GGNN) in the SE-SG Embedding Module captures the hidden state of students based on SE-SG. Learning interactive information related to students' exercise and answering behavior is acquired through the self-attention module with a forgetting curve mechanism for exercise and skill embedding representations. Subsequently, the Difficulty Analysis Module examines the difficulty characteristics of current exercises based on their inherent features. Finally, the IRT Prediction Module computes the probability of students answering the next exercise correctly. Algorithm 1 outlines the specific steps of the implemented algorithm.

**Algorithm 1:**
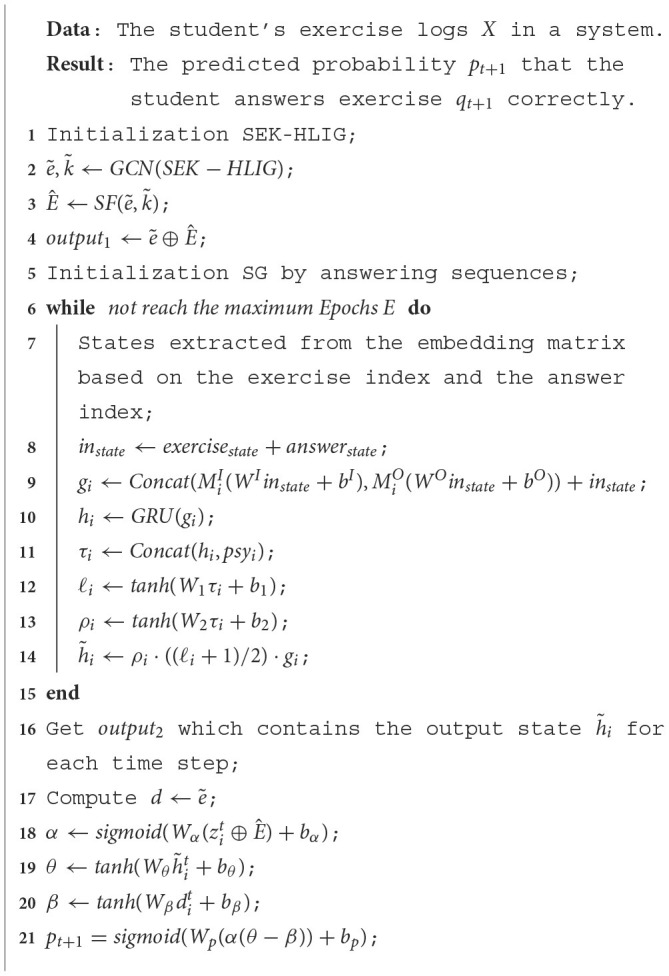
The proposed Psy-KT method for understanding the learning process.

### 4.2 Student-Exercise-Skill Heterogeneous Learning Interactive Graph Embedding Module

This module utilizes a Graph Convolutional Network (GCN) to derive embedding representations for exercises and skills from the Student-Exercise-Skill Heterogeneous Learning Interactive Graph (SEK-HLIG). In the SEK-HLIG, neighbor nodes of an exercise consist of exercises that share the same skill or other exercises answered by the same student. Similarly, the neighbor nodes of a skill comprise other skills used by the exercise that employs the skill. The GCN leverages these interaction paths defined in SEK-HLIG to process the obtained information. The dissemination process involves two types of matrices: an “exercise-exercise” matrix, encompassing interaction paths for exercises sharing the same skill or answered by the same student, and an “exercise-skill matrix,” including interaction paths for other skills used in the exercise that employs the skill.

In the context of the Student-Exercise-Skill Heterogeneous Learning Interactive Graph Embedding Module, it is evident that all three interaction paths discussed are linked to the exercise node. Consequently, the nodes along these paths associated with an exercise can collectively be termed as the neighboring nodes of that exercise. The aggregation process involves combining the exercise node with its neighboring nodes. Notably, student nodes are excluded from this aggregation process; their role is solely to facilitate connections in the SEK-HLIG interactions.

**Figure 4:**
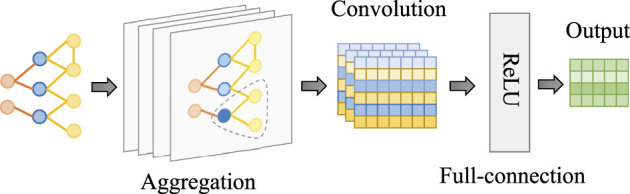
Schematic structure of the GCN network. Comprising three key components: the aggregation layer, convolutional layer, and fully connected layer, it ultimately outputs high-dimensional information for exercises and skills.

The structure of the Graph Convolutional Network (GCN) is visually represented in Figure 4. Upon inputting the interaction path from SEK-HLIG into the GCN, the model aggregates neighbor information to the node by traversing multiple layers. Subsequently, it computes the output high-dimensional information ($ẽ,\tilde{k}$) for exercises and skills through the convolutional layer and the fully connected layer.

Within the convolutional layer of the GCN, the feature information of the *i*th node in the graph, which represents an exercise node *e*_*i*_ or a skill node *k*_*i*_, is denoted as *z*_*i*_. The forward propagation equation for this node is expressed in Equation (5).


(5) $$\begin{array}{l} z_{i}^{\left(l\right)}=\sigma \left(\underset{j\in i}{\sum }W^{l-1}z_{j}^{l-1}+b^{l-1}\right) \end{array}$$

Here, σ signifies the nonlinear activation function ReLU, while *W*^*l*−1^ and *b*^*l*−1^ represent the weight matrix and bias vector, respectively. This equation encapsulates the fundamental process by which the GCN's convolutional layer operates, propagating feature information of nodes within the graph.

### 4.3 Student-Exercise Sequential Graph Embedding Module

**Figure 5:**
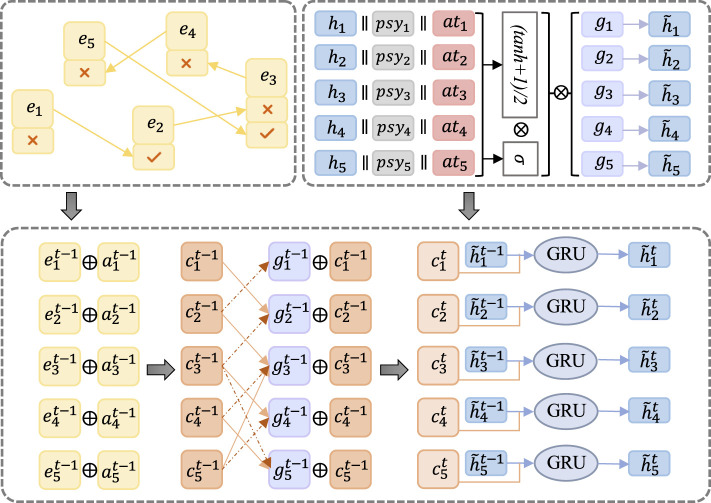
Schematic structure depicting the student's hidden state through the GGNN network. The learning sequence is abstracted into a graph structure, where information is extracted and merged. The resulting output from the GGNN network serves as the student's hidden features.

This module employs the Gated Graph Neural Network to derive the hidden state of students based on the Student-Exercise Sequential Graph (SE-SG). The GGNN processes feature information, and the corresponding schematic structure is illustrated in Figure 5.

Within the SE-SG, node information is computed to obtain the merged result *r*_*i*_ using Equation (6).


(6) $$\begin{array}{l} r_{i}=e_{i}⊕a_{i} \end{array}$$

Here, ⊕ denotes the bitwise summation, while *e*_*i*_ and *a*_*i*_ represent an exercise and its corresponding answer in SE-SG, respectively.

The computation of the new state representation *g*_*i*_ is derived from the merged node information *r*_*i*_. Given that SE-SG is a directed graph, both the incoming and outgoing edges of a node are considered in this process. The formalized expression for *g*_*i*_ is defined in Equation (7).


(7) $$\begin{array}{l} g_{i}=concat\{M_{i}^{I}\left(\left[r_{1},r_{2},\ldots ,r_{n}\right]W^{I}+b^{I}\right),\\ \;M_{i}^{O}\left(\left[r_{1},r_{2},\ldots ,r_{n}\right]W^{O}+b^{O}\right)\} \end{array}$$

Here, $M_{i}^{I},M_{i}^{O}\in {\text{R}}^{1\times n}$ represents the corresponding row *i* in the exercise-answer matrix, *W*^*I*^, *W*^*O*^∈ℝ^*d*×*d*^ are the weight matrices, and *b*^*I*^, *b*^*O*^∈ℝ^*d*^ are the bias vectors.

### 4.4 Knowledge State Modeling Module

The process of updating the hidden knowledge state, denoted as *h*_*i*_, involves the Gated Recurrent Unit (GRU) unit, where relevant information, including psychological features (*psy*_*i*_) and time spent by the student in answering an exercise (*at*_*i*_), is incorporated. The resulting new hidden knowledge state, ${\tilde{h}}_{i}$, is computed through a series of equations. Initially, the relevant state information is combined to form τ_*i*_ in Equation (8).


(8) $$\begin{array}{l} \tau_{i}=concat\left\{h_{i},psy_{i},at_{i}\right\} \end{array}$$

Subsequently, the learning gains are formalized in Equation (9), recognizing that not all learning gains lead to an increase in student knowledge. Equation (10) is introduced to control the quantity of knowledge that students acquire:


(9) $$\begin{array}{l} \ell_{i}=tanh\left({W_{1}\tau }_{i}+b_{1}\right) \end{array}$$

(10) $$\begin{array}{l} \rho_{i}=sigmoid\left(W_{2}\tau_{i}+b_{2}\right) \end{array}$$

Here, $W_{1},W_{2}\in {\mathbb{R}}^{d\times d}$ represent the weight matrices, and $b_{1},b_{2}\in {\mathbb{R}}^{d}$ represent the bias vectors.

The computation of ${\tilde{h}}_{i}$ is expressed in Equation (11). To ensure that ℓ_*i*_ remains positive, a linear transformation is applied since the range of tanh is (−1, 1). The learning gain at a specific timestamp is obtained by multiplying ℓ_*i*_ and ρ_*i*_, and the overall learning gain is derived by multiplying with *g*_*i*_:


(11) $$\begin{array}{l} {\tilde{h}}_{i}=\rho_{i}\cdot \left(\left(\ell_{i}+1\right)/2\right)\cdot g_{i} \end{array}$$

In the Gated Graph Neural Network, the hidden knowledge state ${\tilde{h}}_{i}$ is updated iteratively, with *g*_*i*_ serving as input to each GRU unit, and ${\tilde{h}}_{i}$ as the output. The node information is updated using Equation (12):


(12) $$\begin{array}{l} r_{i}^{t}=r_{i}^{t-1}⊕g_{i}^{t-1} \end{array}$$

The calculation of the Gated Recurrent Unit (GRU) unit involves several distinct steps to update the hidden state at each time step. These steps are outlined as follows:

1. Reset gate: This gate determines how the previous hidden state should be considered at the current time step and is formalized in Equation (13).

(13) $$\begin{array}{l} \omega_{i}^{t}=sigmoid\left(W_{\omega }\cdot \left[r_{i}^{t},{\tilde{h}}_{i}^{t-1}\right]\right) \end{array}$$

2. Update gate: The update gate dictates how to combine the new candidate hidden state with the current hidden state to update it for the current time step. The computation is expressed in Equation (14).

(14) $$\begin{array}{l} \xi_{i}^{t}=sigmoid\left(W_{\xi }\cdot \left[r_{i}^{t},{\tilde{h}}_{i}^{t-1}\right]\right) \end{array}$$

3. Candidate hidden state: This is a temporarily calculated value serving as an intermediate result for updating the hidden state in Equation (15).

(15) $$\begin{array}{l} \gamma_{i}^{t}=tanh\left(W_{\gamma }\cdot \left[r_{i}^{t},\left(\omega_{i}^{t}⊙{\tilde{h}}_{i}^{t-1}\right)\right]\right) \end{array}$$

4. Update hidden state: This step is responsible for updating the hidden state and is computed in Equation (16).

(16) $$\begin{array}{l} {\tilde{h}}_{i}^{t}=\left(1-\xi_{i}^{t}\right)⊙{\tilde{h}}_{i}^{t-1}+\xi_{i}^{t}⊙\gamma_{i}^{t} \end{array}$$

Here, $W_{\omega },W_{\xi },W_{\gamma }\in {\mathbb{R}}^{d\times 2d}$ denote parameters trainable by the model, and ⊙ denotes the inner product. These sequential computations collectively facilitate the propagation of information between nodes in the GRU, following the underlying graph structure.

### 4.5 Forgetting Module

The preceding SEK-HLIG Embedding Module, utilizing Graph Convolutional Networks (GCN), has successfully obtained embedded representations of exercises and skills. However, the intricacies of the student learning process extend beyond mastering skills and linking exercises to relevant skills; the phenomenon of forgetting after learning is a crucial aspect of semantic information. This module is introduced to capture semantic details related to students' exercise-answering processes, aiming to enrich the embedded representations of exercises and skills and provide a more comprehensive understanding of students' learning journeys.

Within this module, the forgetting function is expressed by Equation (17):


(17) $$\hat{E}=\left\{f_{i}\left(a*exp^{-b*x}+c\right)|i\in 1,2,\ldots ,t-1\right\}$$

Here, Ê signifies the new student answering state calculated by the forgetting function, with *a, b, c* being learnable parameters, and *x* representing the interval between the *i*th timestamp and the initial timestamp. *f*_*i*_∈*F* denotes the answering state of the student at a specific timestamp, which can be computed using Equation (18):


(18) $$\begin{array}{l} A=\frac{\left(EW^{Q}\right){\left(EW^{K}\right)}^{T}}{\sqrt{b^{k}}} \end{array}$$

In this equation, *A* represents the attention matrix, *W*^*Q*^, *W*^*K*^, *W*^*V*^ are weight matrices, *E* = {*e*_*i*_|*i*∈[1, 2, …, *t*−1]} corresponds to the student's responses to exercises, and $\sqrt{b^{k}}$ acts as the scaling factor, ensuring stability in the attentional weights by balancing their scaling. The expression of *E* enables the calculation of correlations between different components, aiding in determining the critical elements for the task.

The attention weights are utilized to compute the softmax function in Equation (19):


(19) $$\begin{array}{l} F=softmax\left(A\left(EW^{V}\right)\right) \end{array}$$

Once the attention weight matrix *A* is determined, it is applied to the value matrix *EW*^*V*^ to derive the final feature representation of the student's answering state. This module plays a crucial role in capturing the temporal dynamics of learning, specifically addressing the phenomenon of forgetting, and contributes to a more nuanced understanding of the evolving knowledge states of students.

### 4.6 Difficulty Analysis Module

In real-world educational scenarios, the complexity of individual exercises tends to vary significantly, exerting a notable influence on students' proficiency in answering them accurately. The difficulty of an exercise is intrinsic to the exercise itself and remains independent of the student's mastery of corresponding knowledge and skills. Therefore, the exercise difficulty is determined by leveraging the feature information of the nodes in SEK-HLIG, as outlined by Equation (20):


(20) $$\begin{array}{l} d_{i}=W_{d}z_{i}+b_{d} \end{array}$$

In this equation, *W*_*d*_ represents the weight matrix, *b*_*d*_ denotes the bias vector, and *z*_*i*_ encapsulates the feature information of the *i*th node. This calculation ensures that the exercise difficulty is solely derived from the inherent characteristics of the exercise, untethered from individual student competencies in the corresponding knowledge and skills. The Difficulty Analysis Module provides a crucial mechanism for objectively assessing and categorizing the complexity of exercises, contributing to a more nuanced understanding of the educational environment.

### 4.7 IRT Enhanced Prediction Module

The Item Response Theory model, rooted in psychological theory, explores the correlation between students' abilities and the accuracy of their responses. This model incorporates three key parameters, which are exercise differentiation coefficient (α), student's ability (θ), and exercise difficulty (β), calculated through Equations (21)–(23).


(21) $$\begin{array}{ll} \alpha & =sigmoid\left(W_{\alpha }\left(z_{i}^{t}⊕Ê\right)+b_{\alpha }\right) \end{array}$$

(22) $$\begin{array}{ll} \theta & =tanh\left(W_{\theta }{\tilde{h}}_{i}^{t}+b_{\theta }\right) \end{array}$$

(23) $$\begin{array}{ll} \beta & =tanh\left(W_{\beta }d_{i}^{t}+b_{\beta }\right) \end{array}$$

Here, *W*_α_, *W*_θ_, and *W*_β_ are learnable weight parameters, while *b*_α_, *b*_θ_, and *b*_β_ are learnable bias parameters. The exercise differentiation coefficient (α) reflects the quiz exercise's ability to distinguish the student's level, dependent not only on the student's ability but also intricately tied to the exercise itself. This parameter is defined through the node feature information $z_{i}^{t}$. The enhanced student answering state (Ê) captures semantic information gleaned from the process of answering exercises. The student's ability (θ) is defined exclusively through the student's hidden knowledge state ${\tilde{h}}_{i}^{t}$. The exercise difficulty (β) is considered solely in relation to the exercise and is defined through *d*_*i*_ calculated by the difficulty analysis layer.

According to IRT theory, the probability *p*_*t*+1_ that a student correctly answers an exercise in the subsequent timestamp is calculated by Equation (24):


(24) $$\begin{array}{l} p_{t+1}=sigmoid\left(W_{t+1}\left[\left(\alpha \left(\theta -\beta \right)\right)\right]+b_{t+1}\right) \end{array}$$

Here, *W*_*t*+1_ and *b*_*t*+1_ are learnable parameters. *p*_*t*+1_∈[0, 1], and when *p*_*t*+1_∈[0, 0.5], the student is deemed to have answered incorrectly, while *p*_*t*+1_∈(0.5, 1] indicates a correct response.

To optimize the model, the loss function is defined as Equation (25), where *y*_*t*+1_ denotes the actual label and *p*_*t*+1_ denotes the model-predicted result:


(25) $$\begin{array}{l} Loss=-\underset{t}{\sum }\left[y_{t+1}log\left(p_{t+1}\right)+\left(1-y_{t+1}\right)log\left(1-p_{t+1}\right)\right] \end{array}$$

Minimizing this loss function aims to enhance the model's predictive accuracy for diverse exercises, thereby improving the overall learning process performance. This optimization facilitates the personalized education system in better understanding and adapting to individual student states, enabling more effective learning support and guidance.

## 5 Experiments

This section outlines the experiments conducted to evaluate the Psy-KT model's performance in comparison to five existing knowledge tracing models across four publicly available datasets. The overarching goal is to substantiate the efficacy of the proposed framework in the knowledge tracing task. The experiments seek answers to four Research Questions (RQ), elucidating different facets of the model's performance and contributing to a comprehensive understanding of its strengths and contributions.

- **RQ1:** How does the performance of our proposed Psy-KT model compare to state-of-the-art KT methods?
- **RQ2:** What is the significance of introducing psychological factors to model learning performance in the Psy-KT model?
- **RQ3:** What is the impact of incorporating the forgetting function into the Psy-KT model on modeling the learning process?
- **RQ4:** Is the Item Response Theory effective in making learning performance predictions, and how should it be interpreted?

### 5.1 Datasets

This section outlines the datasets used in our experiments, emphasizing their characteristics and the rationale behind their selection. Four public datasets were employed, each serving a specific purpose in evaluating the Psy-KT model. Two datasets included psychological indicators, allowing us to investigate the impact of such factors on students' responses. To assess the model's performance in the absence of psychological indicators, two additional datasets without such features were included for comparative analysis. Table 2 presents key statistics for these datasets.

**Table 2:** Statistics of the datasets used for the experiments.

**Items**	**Assist12**	**Assist17**	**Assist09**	**Algebra05**
Number of students	28,834	1,709	4,151	174
Number of skills	245	102	138	26
Number of exercises	50,988	3,162	16,891	1,021

**Assist2012–2013:** This dataset comprises student exercise data collected on the ASSISTments platform during the 2012–2013 school year. It includes psychological factors such as average frustration level, confusion level, concentration level, and boredom level during exercise answering (Wang et al., 2015). In our work, we processed this dataset by treating skills as null and removing erroneous data (e.g., negative answer times). After processing, the dataset consists of 28,834 students, 245 skills, and 50,988 exercises, along with corresponding data on answer times and psychological factors.

**Assist2016–2017:** Collected in 2017 on the ASSISTments platform, this dataset provides additional data related to psychological factors. We selected mean values from this data as features. Similar to Assist2012–2013, the dataset was processed, resulting in 1,709 students, 102 skills, and 3,162 exercises, along with data on answer times and psychological factors.

**Assist2009–2010:** Collected during the 2009–2010 school year on ASSISTments, this dataset lacks psychological factor data and serves as a control. Processed similarly to Assist2012–2013, it comprises 4,151 students, 138 skills, and 16,891 exercises.

**Algebra2005–2006:** Provided by the Carnegie Corporation, this dataset contains data generated by students in a math course (Lalwani and Agrawal, 2019). Similar to Assist2009–2010, it serves as a control and lacks psychological factors. After processing, the dataset consists of 174 students, 26 skills, and 1,021 exercises.

Our study is centered on integrating psychological factors into the modeling of learning processes. Consequently, the accuracy and objectivity with which psychological features are collected in the datasets are critical to this research. The psychological factor data in the ASSISTment dataset is collected through the construction of an emotion detector (San Pedro et al., 2013), which encodes students' emotional or behavioral states such as boredom, frustration, engaged concentration, confusion, off-task behavior, gaming, or any other arbitrary states (Pardos et al., 2013). The construction of this emotion detector is divided into two parts: first, observations of students are made using an Android app and their states are labeled, and then these labels are used to create an automated emotion detector that can be applied to large-scale log files. During the observation phase, two coders simultaneously coded the same student and achieved good consistency (compared to random; San Pedro et al., 2013). Furthermore, the ASSISTment dataset has been extensively applied across numerous studies, encompassing a broad range of areas such as psychometrics, learning analytics, personalized education, and the evaluation of teaching effectiveness. This widespread use underscores its significance and impact within the domains of psychological and educational research, establishing it as a widely acknowledged and utilized data resource.

In summary, these datasets collectively provide a diverse and comprehensive foundation for evaluating the performance of the Psy-KT model under different contexts and conditions, enabling us to draw meaningful conclusions about its effectiveness.

### 5.2 Comparison methods

In this section, we present the comparison methods employed in our study, comprising five deep learning-based knowledge tracing models DKT, DKT+, KPT, AKT, and DKVMN, and three graph structure-based knowledge tracing models GKI, GIKT, and SGKT. The primary aim of this comparative experiment is to assess and contrast the performance of our proposed model against the three aforementioned graph structure-based models. The evaluation seeks to identify which model demonstrates superior efficacy in the knowledge tracing task.

1. **DKT:** The DKT model (Piech et al., 2015) marks a groundbreaking foray into applying deep learning to knowledge tracing. Leveraging recurrent neural networks, it captures students' responses, utilizes numerous artificial neurons to delineate temporal dynamics, and extracts potential knowledge states from the data. The paper presents two types of recurrent neural networks: a conventional sigmoid-based RNN and an LSTM model. For our comparison experiment, we opted for the RNN-based variant. The requisite dataset comprises three columns: student ID, skill ID corresponding to the exercise, and an indicator denoting correct or incorrect answers. Each row encapsulates a piece of answer data.
2. **DKT+:** The DKT+ model, proposed in Yeung and Yeung (2018), improves upon some issues present in the DKT model. This model adds three regularization terms to the loss function of the DKT algorithm to address issues of fluctuation and reconstruction, while also considering the current interaction. The dataset for this model comprises a triad, denoted as *X* = *que*_*num*_, *E, A*, where *que*_*num*_ is the number of exercises answered, *E* denotes exercise IDs, and *A* denotes the set of responses to each exercise. Each triad represents a sequence of answers for a student.
3. **KPT:** The Knowledge Proficiency Tracing (KPT) model is proposed based on matrix factorization. This model first associates each exercise with a skill vector. Given the student's exercise feedback log and the Q-matrix (representing the relationship between exercises and skills), KPT utilizes the Q-matrix to map each student's latent skill vector into the skill space. It combines the prediction of students' performance in the next time step based on the learning curve and forgetting curve. The input data for this model consists of two parts: the Q-matrix, which represents the relationship between exercises and skills, and the student's answer data information. The student's answer data information is represented as *X* = *studentId, ProblemId, Answer*, containing the unique identifier of the student and the exercises attempted by the student along with their corresponding answers.
4. **AKT:** Due to the limitation of RNN used in DKT to handle excessively long input sequences, an AKT model was proposed. This model, regardless of input sequence length, directly captures the relevance of each item in the input to obtain a global relationship. The dataset required for AKT is a quaternion, denoted *X* = *que*_*num*_, *E, K, A*, containing the number of exercises that have been answered, the set of exercise numbers associated with those exercises, the skill numbers, and the answers to each exercise. Each quaternion represents a sequence of student answers.
5. **DKVMN:** The Dynamic Key-Value Memory Network (Zhang et al., 2017) employs a static matrix called keys to store knowledge skills and a dynamic matrix called values to store and update mastery levels. These matrices collaborate to reveal the fundamental skills of annotated exercises by students, portraying the evolving knowledge state. The dataset required for this model is in the same format as the dataset required for DKT+ model.
6. **GKT:** The Graph-based Knowledge Tracing model (Nakagawa et al., 2019) utilizes diverse techniques to aggregate neighboring features for updating node features. In our comparison, we employ Dense Graph computation based on a static approach. The updated embedded representation is then used to predict the student's performance at the next step. The required data structure for GKT does not demand extensive processing; it only involves deleting duplicates and null data from the original dataset.
7. **GIKT:** The Graph-based Interaction Knowledge Tracing model (Yang et al., 2021) incorporates Graph Convolutional Networks (GCN) to delineate the relationship between skills and exercises. Introducing a History Recap module and an Interaction module further details the relationships within the student-exercise-skill triad. GIKT organizes the dataset as a quaternion, denoted as *X* = *que*_*num*_, *K, E, A*, encompassing the number of exercises answered, the set of skill numbers associated with those exercises, exercise numbers, and responses to each exercise. Each quaternion represents a sequence of answers for a student.
8. **SGKT:** The Session Graph-based Knowledge Tracing model (Wu et al., 2022) conceptualizes the student answer sequence as a Session Graph. It extracts the student's hidden learning state through Gated Graph Neural Networks and acquires semantic descriptive information through Graph Convolutional Networks (GCN) and a Self-attention mechanism. The amalgamation of these components is then employed for predicting the student's answer. The dataset structure for SGKT aligns with that of the GIKT model.

### 5.3 Evaluation metrics and basic parameterization

In this experiment, we employ several metrics, including AUC, Accuracy, Precision, and F1-Score, to comprehensively evaluate the performance of the models.

1. *AUC*: AUC (Area Under the Curve) measures the area under the Receiver Operating Characteristic (ROC) curve, indicating the area between the curve and the axes. A higher AUC value suggests superior model performance, signifying better discrimination between positive and negative cases. An AUC of 1 indicates perfect classification, while an AUC of 0.5 signifies a model equivalent to random guessing.
2. *Accuracy*: Accuracy assesses the model's ability to correctly categorize samples and is calculated in Equation (26).

(26) $$\begin{array}{l} Accuracy=\frac{TP+TN}{TP+TN+FP+FN} \end{array}$$

where *TP* is the number of samples correctly predicted as positive, *TN* is the number of samples correctly predicted as negative, *FP* is the number of samples incorrectly predicted as positive, and *FN* is the number of samples incorrectly predicted as negative. The numerator, *TP*+*TN*, represents the correctly categorized samples, and the denominator, *TP*+*TN*+*FP*+*FN*, represents the total number of samples.

3. *Precision*_*class*_: To assess the balance of model performance, we use the class-specific Precision. It gauges the proportion of samples predicted by the model as positive, which are indeed positive examples. It is calculated in Equation (27).

(27) $$\begin{array}{l} Precision_{class}=\frac{TP_{class}}{TP_{class}+FP_{class}} \end{array}$$

4. *F*1_*class*_: To assess the balance of model performance, we employ the class-specific F1. It integrates the class-specific Precision and Recall, effectively balancing false positives and false negatives. With a range of 0 to 1, it is calculated using Equation (28).

(28) $$\begin{array}{l} F1_{class}=2\cdot \frac{Precision_{class}+Recall_{class}}{Precision_{class}\cdot Recall_{class}} \end{array}$$

This experiment was conducted on a server equipped with an NVIDIA GeForce RTX 2080 Ti GPU, utilizing the Python 3.8 and TensorFlow 2.4 framework along with the Adam optimizer for model training. The dataset was split, allocating 80% for training and 20% for testing purposes.

The essential parameters for the proposed Psy-KT model, as outlined in this paper, are configured as follows:

- The maximum number of training times is 200.
- The learning rate is set to 0.00025.
- The learning rate decay factor is set to 0.92.
- Different training batches are set according to different datasets. For example, it is set to 6 for the Assist12 dataset and 12 for the Algebra05 dataset.
- The Drop layer parameter was set to [0.8,0.8,1] to prevent overfitting.

### 5.4 Experiment result and analysis

#### 5.4.1 Main results (RQ1)

1. **AUC:** The outcomes of the experiment conducted on four public datasets are summarized in Table 3. The Area Under the Curve (AUC) results demonstrate that SGKT exhibits superior performance among existing knowledge tracing models, consistently delivering commendable results across all datasets, with a peak achievement of 81.4% on the Assist12 dataset. The Psy-KT model proposed in this paper outperforms other models in terms of AUC. Across all four datasets, Psy-KT consistently yields slightly higher AUC values than the SGKT model. Specifically, Psy-KT exhibits a noteworthy improvement of 1.7% on the Assist12 dataset, a substantial 3.1% increase on the Assist17 dataset, a 1.3% gain on the Assist09 dataset, and a 0.5% enhancement on the Algebra05 dataset. The Incomplete Gamma Function IRT model demonstrates a modest increase of 0.2% in AUC.

**Table 3:** The performance comparation beween the proposed Psy-KT and the state-of-the-art (SOTA) methods in terms of AUC (%) on four databases.

**Methods**	**Datasets**			
	**Assist12**	**Assist17**	**Assist09**	**Algebra05**
DKT	72.6	66.2	71.2	72.7
DKT+	73.4	65.7	73.5	61.6
KPT	67.2	75.2	75.6	73.2
AKT	73.0	73.8	69.6	75.7
DKVMN	74.6	63.9	73.1	74.8
GKT	75.7	76.2	72.9	73.9
GIKT	77.4	74.5	78.5	75.1
SGKT	81.4	76.5	79.6	77.4
Psy-KT	**83.1**	**79.6**	**80.9**	**77.6**

Bold indicates the best performance, and underline indicates the second-best performance.

**Figure 6:**
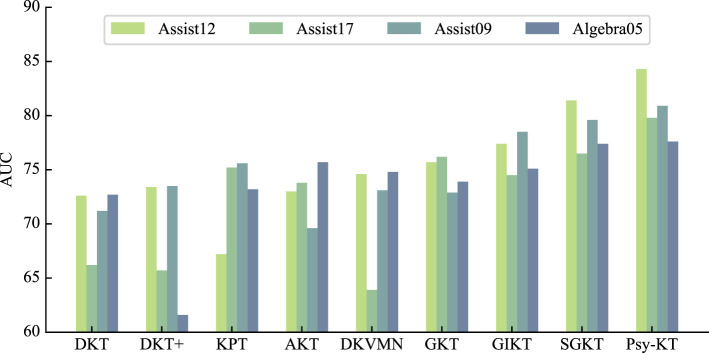
Visual comparison of AUC performance of the method proposed in this paper with the SOTA methods on four databases.

Moreover, as visually represented in Figure 6, distinct performance disparities emerge between datasets. Notably, the AUC of the Psy-KT model exhibits more pronounced improvements in datasets with emotional elements, such as Assist12 and Assist17. This suggests that incorporating emotional elements enhances the model's ability to characterize students' learning processes.

2. **Accuracy:** The accuracy outcomes for each dataset are presented in Table 4. The results indicate that SGKT outperforms the GKT and GIKT models, securing the highest accuracy on the Assist12 dataset. The proposed model exhibits superior accuracy, surpassing SGKT in performance. Specifically, there is a 0.2% improvement on the Assist12 dataset, a substantial 3% increase on the Assist17 dataset, a significant 5.3% gain on the Assist09 dataset, and a 0.4% enhancement on the Algebra dataset.

**Table 4:** The performance comparation beween the proposed Psy-KT and the SOTA methods in terms of Accuracy (%) on four databases.

**Methods**	**Datasets**			
	**Assist12**	**Assist17**	**Assist09**	**Algebra05**
DKT	70.3	65.2	68.7	62.8
DKT+	71.2	66.1	66.0	68.9
KPT	70.3	69.1	71.1	68.4
AKT	73.7	66.5	70.7	67.0
DKVMN	72.5	65.5	68.6	63.4
GKT	72.4	70.4	68.9	62.1
GIKT	72.8	72.9	71.2	70.2
SGKT	78.3	71.3	71.8	76.8
Psy-KT	**78.5**	**74.3**	**76.5**	**77.2**

Bold indicates the best performance, and underline indicates the second-best performance.

**Figure 7:**
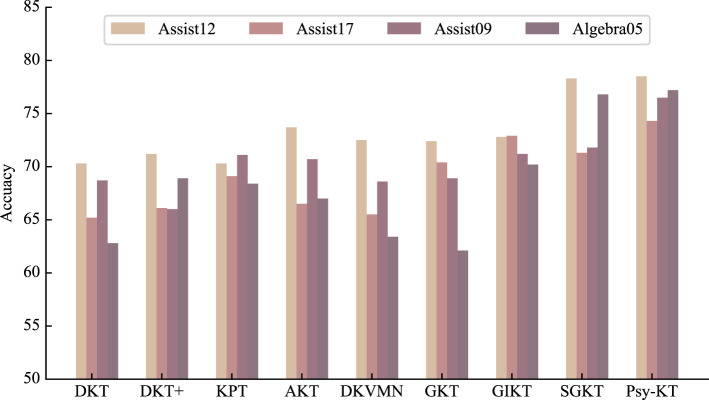
Visual comparison of Accuracy performance of the method proposed in this paper with the SOTA methods on four databases.

Furthermore, as visually depicted in Figure 7, the accuracy of the Psy-KT model remains relatively stable across both types of datasets. This stability suggests that the model adeptly integrates both psychological and non-psychological information, showcasing flexibility in adapting to diverse data types. The model's capacity to achieve stable accuracy underscores its capability to avoid overreliance on psychological factors alone for classification.

3. **Class-specific precision:** The class-specific precision outcomes for each dataset are displayed in Table 5. Examining Class 0 precision across all datasets, Psy-KT consistently achieves the highest precision, indicating its exceptional performance in this category and a high correctness rate. For Class 1 precision, Psy-KT attains the highest precision on the Assist09 dataset.

**Table 5:** The performance comparison between the proposed Psy-KT and the SOTA methods in terms of class-specific precision (%) on four databases.

**Methods**	**Datasets**							
	**Assist12**		**Assist17**		**Assist09**		**Algebra05**	
	**Class 0**	**Class 1**	**Class 0**	**Class 1**	**Class 0**	**Class 1**	**Class 0**	**Class 1**
DKT	61.3	75.6	63.5	60.6	67.5	71.5	63.8	64.0
DKT+	64.9	77.6	68.9	67.9	66.5	72.7	63.1	61.0
KPT	63.4	76.4	71.2	66.2	69.7	74.7	63.0	65.6
AKT	68.3	77.5	73.1	**74.0**	73.6	74.5	65.8	65.4
DKVMN	60.4	73.8	66.3	60.0	68.4	71.1	59.7	64.7
GKT	62.9	75.1	65.2	67.8	68.6	67.4	59.9	65.2
GIKT	64.6	74.4	74.6	69.2	74.7	70.3	62.1	79.4
SGKT	74.5	**79.2**	72.7	67.7	72.3	71.7	58.7	**79.7**
Psy-KT	**80.7**	78.0	**75.2**	70.9	**75.7**	**76.9**	**67.4**	78.8

Bold indicates the best performance, and underline indicates the second-best performance.

**Figure 8:**
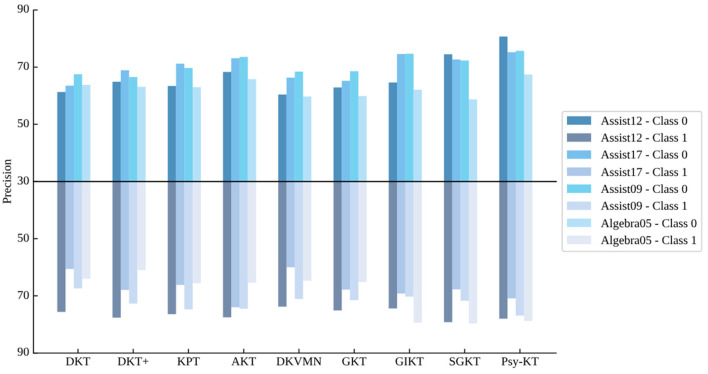
Visual comparison of class-specific precision performance of the method proposed in this paper with the SOTA methods on four databases.

However, as illustrated in Figure 8, SGKT outperforms on the Assist12 and Algebra05 datasets, suggesting that dataset-specific characteristics influence model advantages.

Considering the average precision values for both categories collectively (Table 6), Psy-KT exhibits higher average precision than models without psychological factors (GKT, GIKT, and SGKT) on the Assist12 and Assist17 datasets. This implies that the inclusion of psychological factors significantly enhances performance on these datasets. On the Assist09 and Algebra05 datasets, performance is also improved to a certain extent even in the absence of psychological features. This indicates an overall superior performance and learning ability of the model, allowing it to effectively capture dataset patterns and features even without relying solely on psychological factors.

**Table 6:** The performance comparison between the proposed Psy-KT and the SOTA in terms of average precision (%) on four databases.

**Methods**	**Datasets**			
	**Assist12**	**Assist17**	**Assist09**	**Algebra05**
DKT	68.5	62.1	69.5	63.9
DKT+	71.2	68.4	72.5	69.0
KPT	69.9	68.7	72.1	63.6
AKT	72.9	**73.5**	74.1	65.6
DKVMN	67.1	63.2	69.8	62.2
GKT	69.0	66.5	68.0	62.6
GIKT	69.5	71.9	72.5	70.8
SGKT	76.9	70.2	72.0	69.2
Psy-KT	**79.4**	73.1	**76.3**	**73.1**

Bold indicates the best performance, and underline indicates the second-best performance.

4. **Class-specific F1-Score:** The class-specific F1-Score metrics across different datasets are presented in Table 7. Examining Class 0, SGKT achieves the highest F1-Score on the Assist12 dataset, while the Psy-KT model outperforms on the remaining three datasets. This suggests that the Psy-KT model exhibits a balanced performance on Class 0 when considering Precision and Recall together, resulting in a higher F1-Score. For Class 1, the Psy-KT model attains the highest F1-Score on the Assist12 and Assist09 datasets, showcasing its effectiveness in classifying this category. Both Psy-KT and SGKT achieve the highest F1-Score on the Algebra05 dataset, indicating similar advantages in handling Class 1 for this specific dataset.

**Table 7:** The performance comparison between the proposed Psy-KT and the SOTA methods in terms of class-specific F1-Scores (%) on four databases.

**Methods**	**Datasets**							
	**Assist12**		**Assist17**		**Assist09**		**Algebra05**	
	**Class 0**	**Class 1**	**Class 0**	**Class 1**	**Class 0**	**Class 1**	**Class 0**	**Class 1**
DKT	51.9	72.7	77.5	52.3	60.7	75.3	54.1	77.9
DKT+	54.1	75.2	77.7	52.9	60.6	74.4	57.4	84.1
KPT	56.4	80.0	73.0	54.1	62.8	79.1	54.6	86.6
AKT	58.6	81.2	72.1	56.0	61.1	75.9	58.7	78.3
DKVMN	55.1	75.2	76.3	53.5	60.3	72.8	55.6	79.6
GKT	52.5	78.3	75.4	56.7	55.6	74.9	52.7	82.4
GIKT	53.5	81.9	79.3	**61.0**	59.2	79.6	54.3	85.9
SGKT	**59.4**	85.1	78.3	57.2	60.5	79.5	60.3	**87.8**
Psy-KT	56.7	**85.7**	**82.2**	54.3	**65.7**	**82.1**	**62.3**	**87.8**

Bold indicates the best performance, and underline indicates the second-best performance.

**Figure 9:**
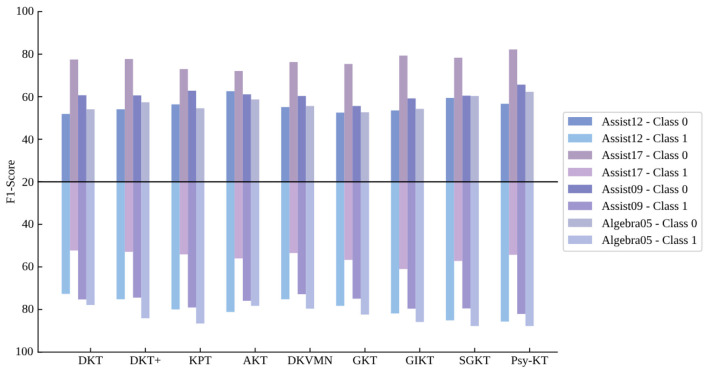
Visual comparison of class-specific F1 performance of the method proposed in this paper with the SOTA methods on four databases.

Notably, as shown in Figure 9, the GIKT model secures the highest F1-Score on the Assist17 dataset, which may be influenced by the dataset's specific nature and label distribution, making the GIKT model more suitable for this type of data.

Considering the average F1-Scores for both categories together (Table 8), the Psy-KT model performs relatively well on the Assist12 dataset with sentiment indicators but slightly lags behind the model without psychological factors on the Assist17 dataset. This suggests that the importance and influence of psychological factors may vary across datasets, leading to performance differences.

**Table 8:** The performance comparison between the proposed Psy-KT and the SOTA methods in terms of Average F1 (%) on four databases.

**Methods**	**Datasets**			
	**Assist12**	**Assist17**	**Assist09**	**Algebra05**
DKT	62.3	64.9	68.0	66.0
DKT+	64.6	65.3	67.5	70.7
KPT	68.2	63.5	70.9	70.6
AKT	69.9	64.1	68.5	68.5
DKVMN	65.2	64.9	66.6	67.6
GKT	65.4	66.1	65.3	67.8
GIKT	67.7	**70.2**	69.4	70.1
SGKT	**72.3**	67.8	70.0	74.1
Psy-KT	71.2	68.3	**73.9**	**75.1**

Bold indicates the best performance, and underline indicates the second-best performance.

The Psy-KT model exhibits commendable performance across various evaluation metrics, notably demonstrating relative strengths in handling datasets enriched with psychological factors. Nevertheless, it is crucial to acknowledge that performance disparities may be influenced by specific characteristics inherent to each dataset, necessitating ongoing refinement and adjustment.

The inclusion of psychological factors in the model proves to be beneficial, providing a more nuanced understanding of students' learning processes. This nuanced perspective enhances the model's effectiveness in predicting students' future performance in answering exercises, showcasing the potential of integrating psychological considerations into knowledge tracing models.

As with any complex model, the Psy-KT's performance is context-dependent, and its optimal utility may vary across different educational datasets. Future work should delve into further refinement and exploration of the model's parameters, considering the intricate interplay between psychological features and diverse dataset characteristics. This iterative process will contribute to a more robust and versatile Psy-KT model, better equipped to handle the nuances of various educational scenarios and student learning contexts.

#### 5.4.2 Convergence rate

**Figure 10:**
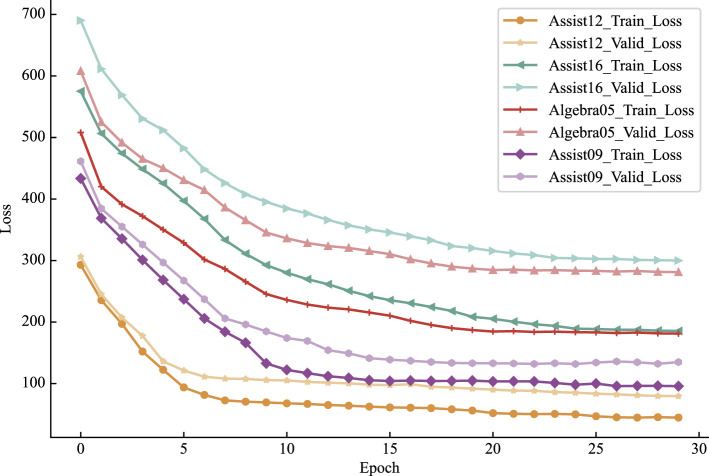
Convergence trends of the Psy-KT model across the four datasets. Although all four datasets exhibit a convergence trend, differences exist in convergence speed and loss magnitude.

This section compares the convergence rates of the Psy-KT model across four distinct datasets to assess its performance. The experimental results are visually represented in Figure 10. Analysis of the outcomes reveals variations in losses across different datasets, with the loss on the validation set generally exceeding that on the training set. Both training and validation set losses exhibit a gradual decline with increasing epochs, suggesting a progressive learning process without apparent signs of significant overfitting.

While all four datasets demonstrate a tendency to converge within different training epochs, there are notable differences in the magnitude of the loss values. These variations may be attributed to factors such as dataset characteristics, size, and the uneven distribution of data. Notably, the validation set losses on the Assist16 and Algebra05 datasets are relatively high in this experiment. This indicates room for improvement in the model's generalization ability for these datasets, highlighting a potential need for additional data preprocessing efforts.

### 5.5 Validity analysis of psychological factors (RQ2)

To ascertain the significance of psychological factors in the knowledge tracing task, an assessment was conducted by excluding the dataset's instances containing psychological factors within Assist12 and Assist17. The model's performance was subsequently evaluated on these modified datasets. The results, depicted in Table 9, reveal that the removal of psychological factors has a nuanced impact on the model's performance metrics.

**Table 9:** AUC and Accuracy (%) of validating the role of psychological factors in knowledge tracing tasks on the Assist12 dataset and the Assist17 dataset, with psychological factors (with psy) and without psychological factors (no psy), respectively.

**Performance**	**Datasets**			
	**Assist12**		**Assist17**	
	**No Psy**	**With Psy**	**No Psy**	**With Psy**
AUC	78.8	**83.1**	70.9	**79.6**
Accuracy	74.2	**78.5**	73.7	**74.3**

Bold indicates the best performance.

**Figure 11:**
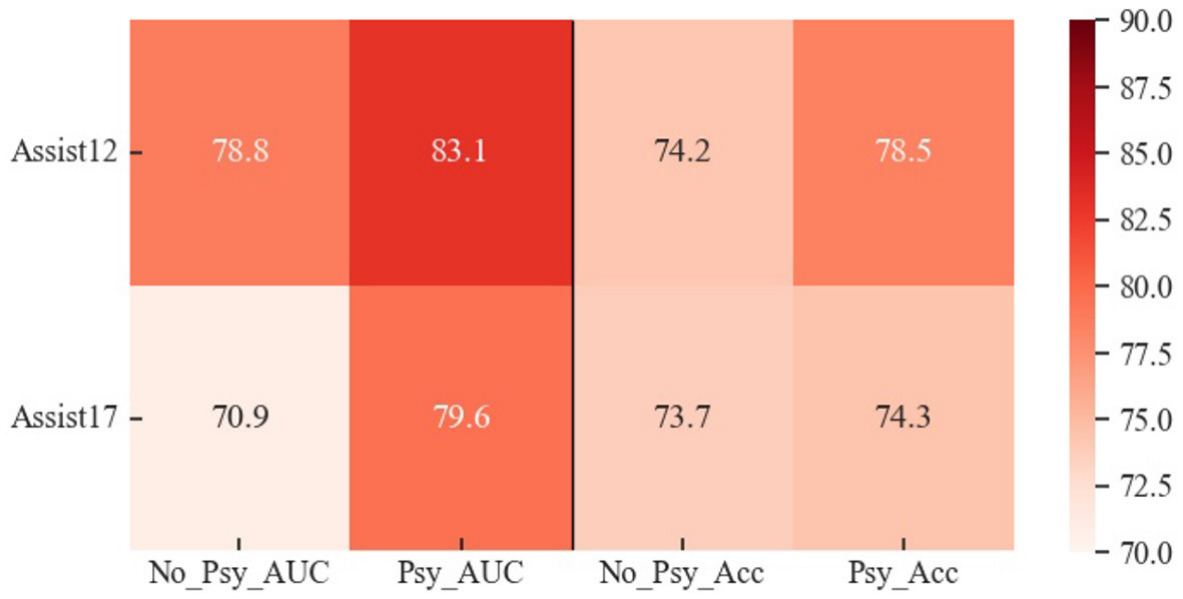
Visual representation of the results of validating the role of psychological factors in the knowledge tracing task on the Assist12 dataset and the Assist17 dataset, with psychological factors (Psy) and without psychological factors (No_Psy), respectively.

Specifically, as shown in Figure 11, there is a significant decrease in all metrics, particularly in AUC, when psychological factors are excluded. Conversely, in datasets that include psychological factors, the model demonstrates improved performance in both AUC and Accuracy.

These findings suggest that the inclusion of psychological factors in the knowledge tracing task positively contributes to the model's ability to describe students' exercise-answering states. This inclusion facilitates a more comprehensive understanding of the students' learning and exercise-answering processes, leading to more accurate predictions of their knowledge tracing. The enhancement in interpretability not only refines the model's predictive capabilities but also enriches its capacity to foresee students' future exercise-answering performance.

### 5.6 Validity analysis of forgetting curve (RQ3)

To assess the impact of the forgetting curve within the Psy-KT model, an alternative model without the forgetting curve was developed. This model was applied to four distinct datasets, and its performance was compared against the original model. The evaluation focused on two key performance metrics, namely Area Under the Curve (AUC) and Accuracy. Results, outlined in Table 10, demonstrate a notable advantage of the model with the forgetting curve across all datasets.

**Table 10:** AUC and Accuracy (%) of validating the role of forgetting curves in a knowledge tracing task on four datasets.

**Performance**		**Datasets**			
		**Assist12**	**Assist17**	**Assist09**	**Algebra05**
AUC	No forget	57.3	61.1	66.9	53.7
		With forget	**83.1**	**79.6**	**80.9**	**77.6**
Accuracy	No forget	67.8	63.0	64.9	61.4
		With forget	**78.5**	**74.3**	**76.5**	**77.2**

Bold indicates the best performance.

The AUC values for the model incorporating the forgetting curve consistently outperform the model without it. This enhancement is particularly evident in the Algebra05 dataset, emphasizing the effectiveness of the forgetting curve in improving the model's performance. Similarly, the model with the forgetting curve exhibits superior Accuracy, with a more pronounced performance gap on the Algebra05 dataset.

**Figure 12:**
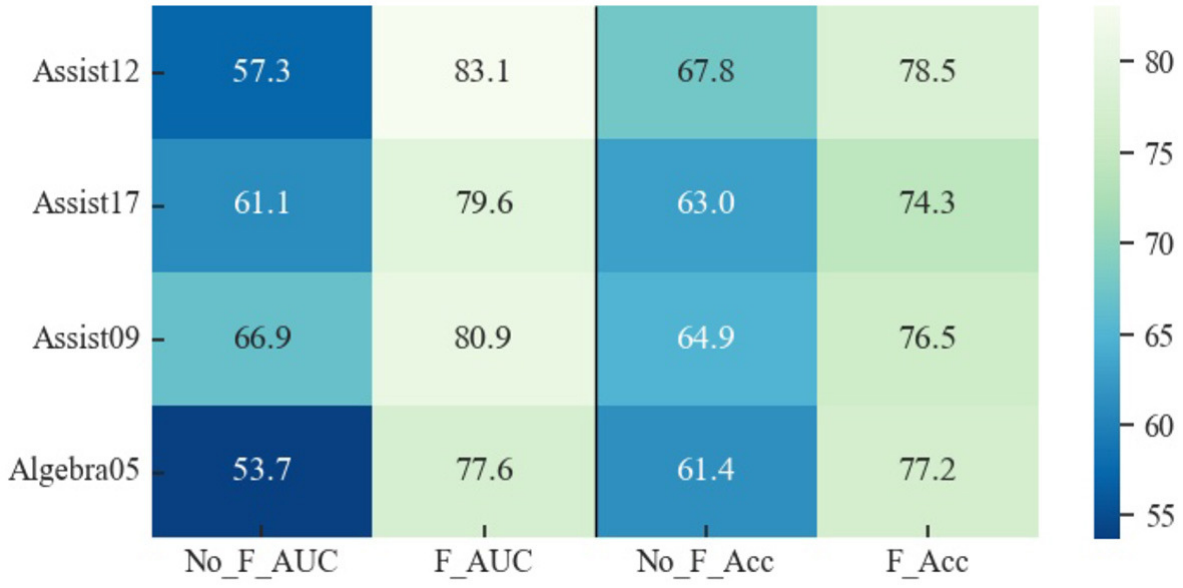
Visual representation of the results validating the role of forgetting curves in a knowledge tracing task on four datasets. No_F_ stands for without forgetting curves for Psy-KT, while F_ stands for Psy-KT with forgetting curves.

While the impact of the forgetting curve varies across datasets, as visually represented in Figure 12, the general trend is a positive correlation between the presence of the forgetting curve and improved model performance. This observation underscores the forgetting curve's utility in simulating the forgetting process inherent in students' learning. Moreover, it contributes to the overall interpretability of the model, aligning with the objective of enhancing its ability to capture the nuances of the forgetting phenomenon.

### 5.7 Validity analysis of IRT enhanced prediction (RQ4)

To scrutinize the validity of the Item Response Theory and its influence on predictive performance, an alternative model utilizing a fully connected layer for predictions is devised. This model is then compared against the IRT theoretical model on diverse datasets, using AUC and Accuracy as evaluation metrics. The results, detailed in Table 11, illuminate the effectiveness of the IRT theoretical model.

**Table 11:** AUC and Accuracy (%) of validating the role of prediction using IRT theoretical models in a knowledge tracing task on four datasets.

**Performance**		**Datasets**			
		**Assist12**	**Assist17**	**Assist09**	**Algebra05**
AUC	FC	54.2	51.3	53.5	51.2
		IRT	**83.1**	**79.6**	**80.9**	**77.6**
Accuracy	FC	67.8	61.1	61.3	60.7
		IRT	**78.5**	**74.3**	**76.5**	**77.2**

FC stands for Psy-KT using fully connected layer for learning prediction, and IRT stands for Psy-KT using IRT for learning prediction. Bold indicates the best performance.

**Figure 13:**
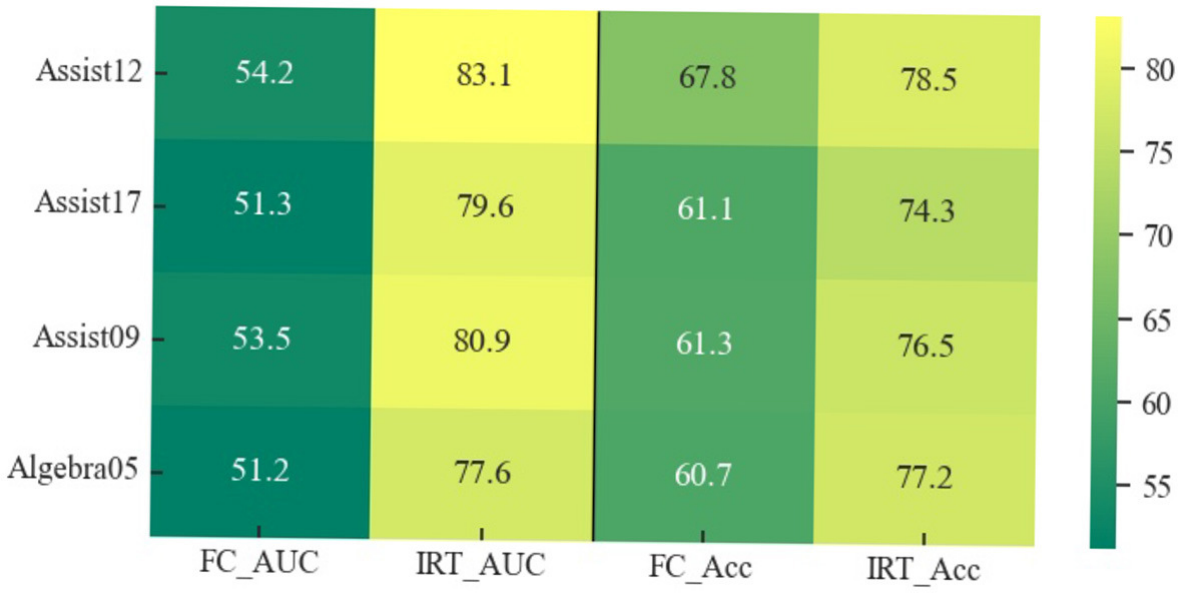
Schematic representation of the results of validating the role of prediction using IRT theoretical models in a knowledge tracing task on four datasets.

The IRT model consistently outperforms the fully connected layer model across all datasets, demonstrating its superior predictive capabilities. Specifically, as visually represented in Figure 13, the AUC values for the IRT model are significantly higher, emphasizing its capacity to better capture the intricate relationship between students' abilities and their exercise-answering performance. The superiority of the IRT model extends to Accuracy as well, reaffirming its effectiveness in predicting students' exercise-answering performance.

These findings suggest that the IRT theoretical model is not only adept at adapting to diverse datasets but also holds substantial potential for practical applications in educational and measurement contexts.

### 5.8 Sensitivity analysis (RQ4)

The IRT theoretical model, incorporating parameters α, β, and θ to quantify student ability, is a cornerstone of the constructed model in this study for evaluating students' future exercise-answering performance. This section aims to elucidate the efficacy of the IRT theoretical model in the context of knowledge tracing. Specifically, a randomly selected exercise from the Assist12 dataset is scrutinized to analyze how the three parameters within the IRT theoretical model articulate student ability.

**Figure 14:**
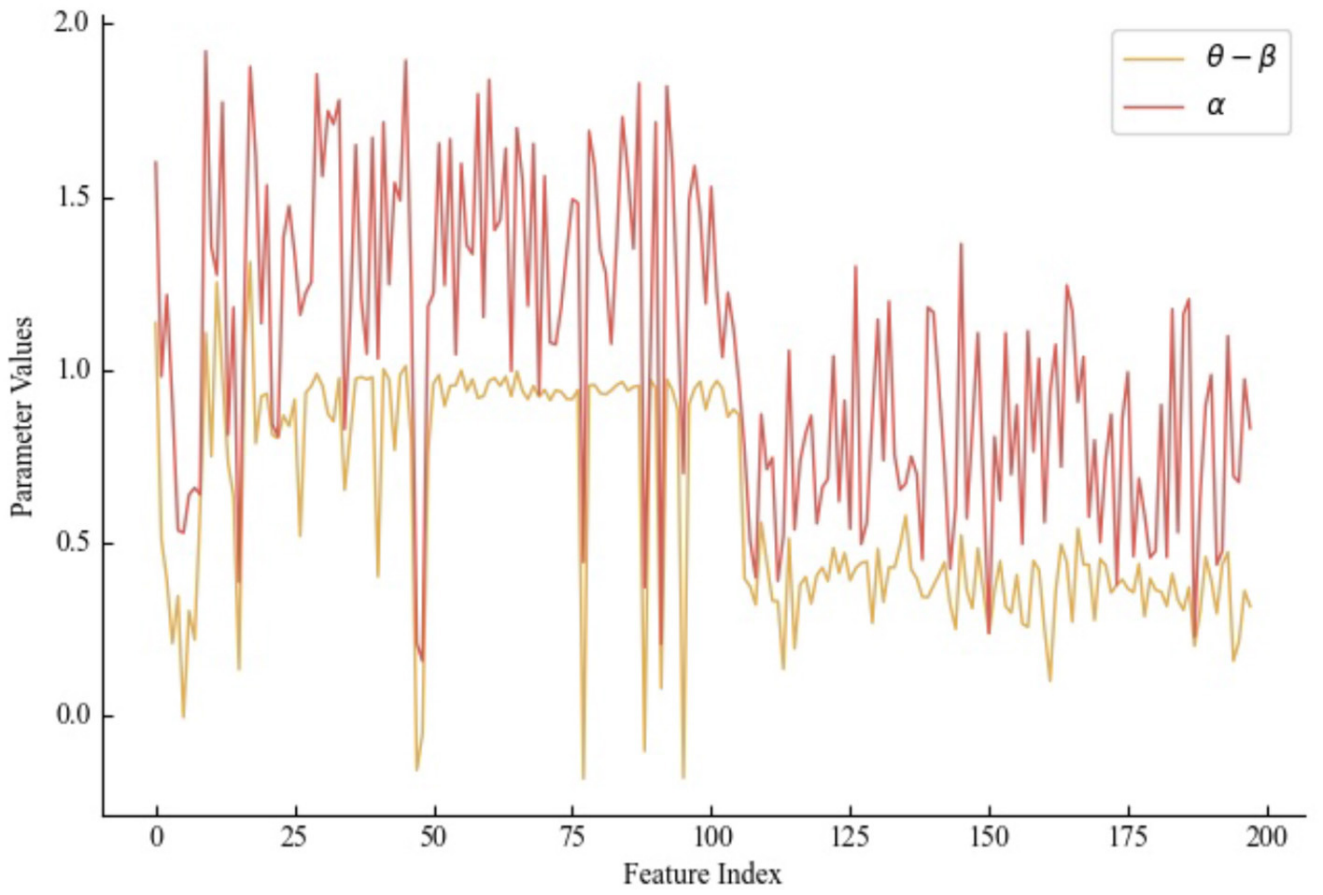
Schematic of the quantization of the three parameters in the IRT model on a randomly selected exercise.

As per the model's prediction, the probability of a student correctly answering the selected exercise is determined to be 0.348. The visualization results of the corresponding three parameters are presented in Figure 14, where the horizontal coordinates denote the multidimensional feature sequences for each parameter, and the vertical coordinates depict the parameter values associated with each feature.

The differentiation parameter α for exercise proficiency fluctuates within the interval [0.15, 1.93], indicating varied sensitivity to different features, reflecting nuanced distinctions in students' abilities. The parameter θ−β gauges the alignment between a student's ability and the exercise difficulty; in several feature dimensions, the student's ability falls below the difficulty valuation (θ−β is a negative value), implying a high likelihood of the student being unable to answer the exercise correctly. In summary, the IRT module adeptly represents students' abilities across different feature dimensions, underscoring the model's effectiveness in knowledge tracing.

### 5.9 Differentiating the proposed model from existing graph-based knowledge tracing models

This section highlights the distinct features and advancements of the Psy-KT model over other graph-based knowledge tracing models, specifically GKT, GIKT, and SGKT, which were used in comparative experiments.

1. **Skill and exercise representation:** GKT, as an initial exploration into graph-based structures for knowledge tracing, utilizes a basic model that primarily captures relationships between skills. Subsequent models, including GIKT and SGKT, advance this by incorporating heterogeneous graphs. In GIKT, nodes represent exercises and skills, explicitly linking skills to exercises. Both the SGKT and our Psy-KT models further enhance this approach by using nodes that represent students, exercises, and skills. This expanded node representation captures complex interactions not only between students and exercises but also between skills and exercises, providing a richer data representation that facilitates a deeper understanding of learning dynamics.
2. **Modeling student states:** The Knowledge State Modeling Module in the Psy-KT model uses a Gated Graph Neural Network (GGNN) that integrates students' historical answer data along with psychological factors. This integration enables a comprehensive view of changes in student states during the exercise answering process. The inclusion of cognitive and psychological data aids in accurately determining students' learning states and tailoring personalized support. In contrast, GKT relies on simpler functions and Multi-Layer Perceptrons (MLPs) to update student states. GIKT uses LSTM along with a module for reviewing historical answers, whereas SGKT employs GGNNs, similar to Psy-KT but without incorporating psychological factors.
3. **Analysis of exercise difficulty:** The Psy-KT model introduces a novel Difficulty Analysis Layer following the construction of the heterogeneous graph and the extraction of embedding representations via a Graph Convolutional Network (GCN). This layer evaluates features that indicate the difficulty level of exercises, thereby enhancing the model's capability to discern varying difficulty levels. This feature distinctly improves the accuracy of predictions concerning student performance, a functionality not available in GKT, GIKT, or SGKT.
4. **Prediction of student performance:** For forecasting students' future performance, Psy-KT integrates an Item Response Theory (IRT) model, which not only increases prediction accuracy but also adds to the interpretability of the results. GKT uses a simpler approach by merging previous outputs through a Sigmoid function. Both GIKT and SGKT extend the modeling of student skills to historical answer records and apply attention networks to compute dual attention weights for all interactions, thus deriving predictions from weighted sums. Psy-KT's use of IRT stands out by providing a more structured and theoretically grounded approach to predictions.

These distinctions underscore the innovative elements of the Psy-KT model, demonstrating its superiority in addressing the complexities of student learning processes through enhanced modeling of interactions and state changes, alongside a thoughtful consideration of instructional content difficulty.

## 6 Conclusions and future work

This paper has introduced the Psy-KT model, a novel approach that enriches knowledge tracing by integrating psychological factors into the analysis of student learning. The model utilizes a heterogeneous learning interactive graph to adeptly capture the complex relationships among students, exercises, and skills. A key innovation of Psy-KT is its incorporation of psychological factors, which offers a more nuanced understanding of students' states during their interactions with learning materials. The model also features a forgetting curve that simulates the natural decay of knowledge over time, thereby enhancing its realism and fidelity. Furthermore, the integration of cognitive parameters and the Item Response Theory model greatly enhances the interpretability and utility of the tracing outcomes.

The performance of the Psy-KT model has been rigorously evaluated across four public datasets, demonstrating its superiority over existing state-of-the-art knowledge tracing models. The inclusion of psychological and forgetting factors notably improves the model's performance, indicating the value of these integrations. Detailed comparative analyzes also affirm the effectiveness of the IRT model within the Psy-KT framework, underscoring its theoretical and practical contributions to the field.

Despite its strengths, the Psy-KT model encounters challenges in performance consistency across different datasets and student groups, highlighting the need for improved robustness and adaptability. The model's reliance on detailed psychological data, which is often limited in availability, poses a significant constraint on the scope of experimental validation and the depth of insight that can be achieved. Addressing the nuanced categorization of psychological factors and mitigating potential overfitting are ongoing challenges.

Looking ahead, future research will focus on enhancing the robustness of the model and expanding the methods for acquiring and integrating psychological data into knowledge tracing. This effort will involve overcoming obstacles related to privacy concerns, data collection methodologies, and the application of advanced data analytics. By navigating these challenges, we aim to further refine the model's accuracy and applicability, thereby contributing more effectively to personalized education strategies and interventions.

## Data availability statement

Publicly available datasets were analyzed in this study. These datasets can be found at: https://sites.google.com/site/assistmentsdata/home/2012-13-school-data-with-affect; https://sites.google.com/view/assistmentsdatamining/dataset; https://sites.google.com/site/assistmentsdata/home/2009-2010-assistment-data; https://pslcdatashop.web.cmu.edu/KDDCup.

## Author contributions

ZW: Conceptualization, Formal analysis, Funding acquisition, Investigation, Methodology, Project administration, Supervision, Validation, Visualization, Writing - original draft, Writing - review & editing. WW: Conceptualization, Data curation, Investigation, Writing - original draft. CZ: Conceptualization, Data curation, Formal analysis, Investigation, Methodology, Project administration, Supervision, Validation, Writing - original draft, Writing - review & editing. HL: Investigation, Validation, Writing - original draft. JS: Investigation, Validation, Writing - original draft.
